# Supplementary material for: Effects of social determinants on children’s health in informal settlements in Bangladesh and Kenya through an intersectionality lens: a study protocol
Source: BMJ Open. 2022 Jun 6;12(6):e056494. doi: 10.1136/bmjopen-2021-056494 (PMC9171224; doi:10.1136/bmjopen-2021-056494)
Supplement: Supplementary data [file bmjopen-2021-056494supp001.pdf]

Supporting information:

Table s1: Description of health outcomes and predictor variables for Nairobi Cross-sectional survey 2012 (NCSS 2012)

Variable name	Description	Categories
**Health outcomes**		
Diarrhea	Whether a child had diarrhea or not in the two weeks preceding the survey	Yes
		No
Fever	Whether a child had fever or not in the two weeks preceding the survey	Yes
		No
Cough	Whether a child had cough or not in the two weeks preceding the survey	Yes
		No
**Predictors**		
**Children demographic characteristics**		
Age	Children up to five were categorised into two groups: “Up to 1 year old (Infants)” and “2-5 years”.	1 year and less (infants)
		2 -5 years
Sex	Sex of a child coded as either male or female	Male
		Female
**Women characteristics**		
Age	Mothers ’age was categorised into two groups: “18 years and less (<18)” and “19 years and above	18 years and under
		19 years and above
**Head of household demographic characteristics**		
Gender	Sex of head of household coded as either male or female	Female
		Male
Ethnicity	Name of ethnic group head of household belongs	Kamba
		Kikuyu
		Luhya
		Luo
		Other
Age	Age of head of household categorised into three categories.	17 – 24years
		25 -34 years
		35 years above
education	Whether head o household has nay education or not	None
		educated
		Don’t know and not applicable

Kibuchi E, *et al. BMJ Open* 2022; 12:e056494. doi: 10.1136/bmjopen-2021-056494

**Social structure**		
Wealth index	Wealth Index were regrouped from five (categories) to three by combining poorest and poorer into one group "poor" and richer and richest into one group "rich" and "Middle".	Rich
		Middle
		Poor
Length of stay	The number of years lived in slums by the household grouped into new migrants (i.e.,2 years and less), old migrants (i.e., more than 2 years), and not applicable/missing.	New migrants
		Old migrants
		Not applicable
Household religion	Name of the religion attended by household	Catholic
		Protestant
		Other
Disability in household	Whether a household has any disabled person residing with them.	Yes
		No
		Missing/Not applicable
Tenure	Does the household own or pay rent to the house they live?	No rent paid
		Pays rent
Food availability	Household's availability of any food in the last 12 months	enough
		not enough
Income generating activity	Household's main source of income	Employed
		Own business
		Not applicable
Health Insurance	Whether a household has a health medical insurance (i.e., either public or private or both)	Yes
		No
health catastrophic costs	Did a household face catastrophic health cost at 40% threshold of in the last 30 days	No
		Yes

Table s2: Description of health outcomes and predictor variables for the analysis for Bangladesh Urban Health Survey 2013 (UHS 2013)

Variable name	Description	Categories
**Health outcomes**		
Acute Respiratory Infection (ARI)	Fever is defined as whether a child had fever or not in the two weeks preceding the survey	Yes
		No
Fever	Whether a child had fever or not in the two weeks preceding the survey	Yes
		No
Cough		Yes

	Whether a child had cough or not in the two weeks preceding the survey	No
**Predictors**		
**Children demographic characteristics**		
Age	Children up to five were categorised into two groups: "Up to 1 year old (Infants)" and "2-5 years".	1 year and less (infants)
		2 -5 years
Sex	Sex of a child coded as either male or female	Male
		Female
**Women characteristics**		
Age	Mothers 'age was categorised into two groups: "18 years and less (<18)" and "19 years and above. Note that the legal age of women at first marriage is 18 years in Bangladesh.	18 years and under
		19 years and above
Ever attended school	Mother ever attended school	Yes
		No
Highest education	Mother's level of education categorised into four groups: "Higher", "Secondary", "Primary", and "No education"	Higher
		Secondary
		Primary
		No education
Marital status	Mother's marital status was categorised into two groups: "Being married", "Not being married"	Being married
		Not being married
Employment	Mothers of respective children was employed last 12 months.	Yes
		No
Religion	Mother's religion was categorized into two groups: "Islam", "Minority religion". Note that Buddhism, Hinduism, Christianism were combined as minority religion in this study.	Islam
		Minority religion
**Head of household demographic characteristics**		
Gender	Sex of head of household was coded as either male or female	Female
		Male
Age	Age of head of household was categorised into three categories.	17 – 24years
		25 -34 years
		35 years above
Marital status	Head of household's marital status was categorised into two groups: "Being married", "Not being married"	None
		educated
		Don't know and not applicable
**Social structure**		
Wealth index	Wealth Index were regrouped from five (categories) to three by combining poorest and poorer into one group	Rich
		Middle

3

	"poor" and richer and richest into one group "rich" and "Middle".	Poor
Length of stay	The number of years lived in slums by the household grouped into new migrants (i.e.,2 years and less), old migrants (i.e., more than 2 years), and not applicable/missing.	New migrants
		Old migrants
		Not applicable
Cooking Fuel used in household	Cooking fuels used in the household were categorized into four: "Charcoal, dung cakes etc.", "Kerosene or liquid gas", "Natural gas", and "Wood fuel"	Charcoal, dung cakes etc.
		Kerosene or liquid gas
		Natural gas
		Wood fuel
Garbage disposal method of households	Garbage disposal method of households were categorized into four: "Disposed within premises", "Collected from home", "Disposed in bin outside", and "Disposed in open spaces"	Disposed within premises
		Collected from home
		Disposed in bin outside
		Disposed in open spaces
Migration status of households	Household's availability of any food in the last 12 months	
Housing Type	Housing type was categorized into two groups: "Multiple story" and "Single story". We collapsed *Jhupri, Mess* as single story.	Multiple story
		Single story
Ownership of the dwelling	Two categories: "yes", "no"	No
		Yes
Ownership of the land	Two categories: "yes", "no"	No
		Yes
Having Separate kitchen	Two categories: "yes", "no"	No
		Yes
Division	Administrative divisions were categorized into four groups: "Dhaka", "Khulna", "Rajshahi" and "Others division". Note that "Barisal", "Chittagong", "Rangpur", and "Sylhet" were combined as "Others division" where ARISE Bangladesh Team conducts research.	Others division
		Dhaka
		Khulna
		Rajshahi

Statistical methodology details

As an illustrative example, consider investigating the effects of child's age (i.e., up to 1 year, 2 to 3 years., 4 to 5 years), child's sex (i.e., female or male), head of household education (none, primary, secondary and higher), sex of the head of household (i.e., male or female), age of the head of household (i.e., 18-25 years, 26-32 years, 33-40 years, >40 years) and household's health insurance status (yes or no). Using the six variables and their corresponding categories: child's age (3), child's sex (2), head of household education (3), head of household sex (2), head of household age (4) and

household's health insurance status (2) we can create 288 intersectional groups/strata. The first stratum can consist of a child who is a female, aged up to 1 year and less, coming from a household whose head is a female, aged 18-25 years and with no education and the household is not covered by health insurance, and the process continues until all the children at level 1 are nested within the groups/strata ($N = 288$) at level 2. This implies that children at level 1 who share similar SDoH factors end up being in the same intersectional group/stratum at level 2.

To capture differences in health disparities in children health outcomes between different groups, for example - males and females, we can assess child's sex-specific effects through their interactions with child 's age, head of household's sex, age and education and household's health insurance using interaction-based fixed model. However, interaction -based fixed effects model formulation only addresses interactions between child's sex and each of the other five variables and not all interactions Eq. 3.

If we were to consider all possible interactions among the six variables, we will have 288 interaction terms in the regression model which may result in the model having parsimony and scalability issues due to geometrical growth of coefficients as more variables are included in the model. In addition, it would be difficult to interpret the results and reduced sample size in some interaction groups may influence whether an association is determined or not (Goldstein, 2011; J. Merlo, 2018; *Supplemental file*). We will overcome this limitation of interaction-based fixed effects models by applying MAIHDA approach (Snijders & Bosker, 2011). This involves treating social strata/groups defined by child's age and sex, head of household sex, age and education and household's health insurance status as strata which will used to explain whether health inequalities are shaped by different characteristics in each stratum.

Let us consider the case where we are interested in investigating effects of child's age (i.e., up to 1 year and less, 2 to 3 years., 4 to5 years), child's sex (i.e., female or male), head of household sex (i.e., male or female), head of household education (none, primary, secondary and higher), head of household age (i.e.,18-25 years, 26-32 years, 33-40 years., >40 years) and household health insurance (yes or no) on children's diarrhea in slums.

Let y_i denote a health outcome of interest (i.e., diarrhea) for child $i(i = 1, \dots, i)$ where,

$$y_i = \begin{cases} 0 \ absence\ of\ diarhea \\ 1 \ Presence\ of\ diarrhea \end{cases} \text{ Eq.(1)}$$

y_i is assumed to follow a Bernoulli distribution, with probabilities $\pi_i = Pr(y_i = 0)$ the probability of child i having no diarrhea and $1 - \pi_i = Pr(y_i = 1)$ the probability of child i having diarrhea. Let X_{1i}represent child sex, X_{2i} represent child's age, X_{3i}represent head of household sex, X_{4i} represent

Kibuchi E, *et al. BMJ Open* 2022; 12:e056494. doi: 10.1136/bmjopen-2021-056494

head of household education, X_{5i} represent head of household age and X_{6i} represent household health insurance. These six variables represent predictor variables. Logistic regression is appropriate for modelling binary (two category) outcomes such as whether a child has diarrhea or not.

The fixed effects logistic regression model for investigating how child sex, child age, head of household sex, head of household education, head of household age and household health insurance are additively associated with child's diarrhea is represented in equation 1 "Eq. (2)".

$$log\left(\frac{\pi_i}{1-\pi_i}\right) = \beta_0 + \beta_1 X_{1i} + \beta_2 X_{21i} + \beta_3 X_{22i} + \beta_4 X_{3i} + \beta_5 X_{41i} + \beta_6 X_{42i} + \beta_7 X_{51i} + \beta_8 X_{52i} + \beta_9 X_{53i} + \beta_{10} X_{6i} \quad \text{Eq. (2)}$$

Eq. (2) estimates the associations of child sex, child age, head of household sex, head of household education, head of household age and household health insurance with child's diarrhea additively (i.e., explanatory effects) and does not accommodate for interactions with each other. In order, to capture specific effects between different groups, for example, child's sex (i.e., males or females), we can assess sex-specific disparities in diarrhea through their interactions with child age, head of household sex, head of household education, head of household age and household health insurance.

Eq. (2) can thus be expanded to include interaction terms, as presented as follows in Eq. (3):

$$log\left(\frac{\pi_i}{1-\pi_i}\right) = \beta_0 + \beta_1 X_{1i} + \beta_2 X_{21i} + \beta_3 X_{22i} + \beta_4 X_{3i} + \beta_5 X_{41i} + \beta_6 X_{42i} + \beta_7 X_{51i} + \beta_8 X_{52i} + \beta_9 X_{53i} + \beta_{10} X_{6i} + \beta_{11} X_{21i} X_{1i} + \beta_{12} X_{22i} X_{1i} + \beta_{13} X_{3i} X_{1i} + \beta_{14} X_{41i} X_{1i} + \beta_{15} X_{42i} X_{1i} + \beta_{16} X_{51i} X_{1i} + \beta_{17} X_{52i} X_{1i} + \beta_{18} X_{53i} X_{1i} + \beta_{19} X_{6i} X_{1i} \quad \text{Eq. (3)}$$

whereβ_{11} to β_{19} are interaction coefficients between child's sex and other explanatory variables in Eq. (2). Not only does Eq. (3) allow for an analysis that considers the association of child's sex in getting diarrhea but also uncovers how other factors that create and sustain diarrhea may differ based on the sex of child. However, Eq. (3) only addresses interactions between child's sex and the other five variables and if we were to consider all possible interactions among the six variables, we will have a total of 288 fixed effects in the logistic model. The higher number of fixed effects may lead to issues with scalability (i.e., a model's inability to accommodate an increase in the number of variables included), model parsimony (i.e., a simple model not having great explanatory predictive power) and reduced sample size in some intersectional groups which may influence whether an effect is determined is determined or not. In addition, it would be difficult to interpret 288 fixed effects. We can overcome these issues by using multilevel analysis of individual heterogeneity and discriminatory accuracy (MAIHDA) approach.

Multilevel analysis of individual heterogeneity and discriminatory accuracy (MAIHDA)

Now, let consider the case where we are interested in investigating effects child's age (i.e., up to 1 year and less, 2 to 3 years., 4 to5 years), child's sex (i.e., female or male), head of household sex (i.e., male or female), head of household education (none, primary, secondary and higher), head of household age (i.e.,18-25years, 26-32years, 33-40 years, >40years) and household health insurance (yes or no) on the outcome diarrhea via intersectionality lens using MAIHDA (Evans, Leckie, & Merlo, 2020; J. Merlo, 2018). This can procced in 3 steps:

The first step involves creating groups/strata based on the categories of the social determinants of health factors (SDoH) we are interested in. This means that children at level 1 who share similar categories SDoH will end up being in the same group/strata at level 2. Therefore, in this example we will have individuals at level 1 nested within 288 groups at level 2.

Therefore, let y_{ij} denote a binary health outcome (i.e., diarrhea) for child $i(i = 1, \dots, n)$ in groups $j\ (j = 1, \dots, N)$ where,

$$y_{ij} = \begin{cases} 0 \ \ absence\ of\ diarrhea \\ 1 \ \ Presence\ of\ diarrhea \end{cases} \quad \text{Eq.(4)}$$

y_{ij} is assumed to follow a Bernoulli distribution, with probabilities $\pi_{ij} = Pr(y_{ij} = 0)$ the probability of child i from stratum/group j having no diarrhea and $1 - \pi_{ij} = Pr(y_i = 1)$ the probability of child i from stratum/group j having diarrhea. Let X_{1ij} represent child sex, X_{2ij} represent child's age, $X_{3ij}j$represent head of household sex, X_{4ij} represent head of household education, X_{5ij} represent head of household age and X_{6ij} represent household health insurance.

The next step involves fitting a null model with children at level one nested within social groups at level two to assess whether there is significant clustering within intersectional strata/groups constructed in step 1. The null model will not include any predictor variables and will only have an intercept to estimate the mean health condition and a random effect to model intersectional strata differences (i.e., variance) and is presented in Eq. 5

$$log\left(\frac{\pi_{ij}}{1-\pi_{ij}}\right) = \beta_0 + \mu_{0j} \quad \text{Eq. (5)}$$

where β_0 is the intercept and $\mu_{0j} \sim N(0, \sigma^2_{group})$ represents the random intercept for the intersectional stratum level residual which is normally distributed with mean 0 and variance σ^2_{μ}. Eq. (5) includes no predictor variables, so the intersectional stratum random effect captures both the main effects of SDoH used to define intersectional strata and their interactions. Assuming no omitted variable bias, the intersectional strata level residual μ_{0j} captures the unique interaction effect for

each intersectional strata (i.e., intersectional -specific differences in health condition) while accounting for sample size differences for each social group. The relevance of the intersectional strata for understanding individual heterogeneity will be evaluated using Variance partitioning Coefficient (VPC) also known as intraclass coefficient (which also informs on the discriminatory accuracy of the intersectional categorisation for distinguishing children with diarrhea from those without (Juan Merlo, Yang, Chaix, Lynch, & Råstam, 2005; Wagner & Merlo, 2013). VPC will be used to quantify the share of the total individual variance in having a health condition that is accounted for at the intersectional strata level with values higher than 5% indicating an acceptable DA (Fisk et al., 2018; Wagner & Merlo, 2013). That is, a high VPC indicates that intersections have a substantially different mean levels of an outcome and that individuals within these group are similar, while a low VPC indicates that individuals within an intersectional group differ substantially. On the other hand, AUC-ROC measures the ability of the model to classify individuals with or without health outcome as a function of individual's predicted probabilities and is and is bounded between 0.5 and 1 (Fisk et al., 2018; Wagner & Merlo, 2013). A value of 0.5 indicates that model predictions are no better than random guessing meaning that predictor variables used in the model have no predictive power, while a value of 1 represents perfect discrimination between under-five with or without health condition (Fisk et al., 2018; Wagner & Merlo, 2013). In our proposed analyses, AUC-ROC values greater than 0.7 and VPC greater than 5% will indicate an acceptable DA and existence of intersectional effects.

The next step involves extending Eq. (5) by can be extended into Eq. (6) by by adjusting for variables used in constructing intersectional strata as fixed effects. Therefore Eq. (6) takes the form:

$$log\left(\frac{\pi_{ij}}{1-\pi_{ij}}\right) = \beta_0 + \beta_1 X_{1ij} + \beta_2 X_{21ij} + \beta_3 X_{22ij} + \beta_4 X_{3ij} + \beta_5 X_{41ij} + \beta_6 X_{42ij} + \beta_7 X_{51ij} + \beta_8 X_{52ij} + \beta_9 X_{53ij} + \beta_{10} X_{6ij} + \mu_{0j} \quad \text{Eq. (6)}$$

where β_0 is the intercept and $\mu_{0j} \sim N(0, \sigma^2_{group})$ represents the group level residual which is normally distributed with mean 0 and variance σ^2_{group}. Assuming no omitted variable bias, the group level residual μ_{0j} captures the unique interaction effect for each social group/strata (i.e. social groups - specific differences in diarrhea) while accounting for sample size differences for each social group. Eq (6) will be used to explore to which extent intersectional strata differences will be explained by SDoH used in constructing intersectional groups. The proportion of variance explained by the adding fixed effects is estimated by calculating the proportional change in variance (PCV) of intersectional strata between a null model defined by Eq. (5) and model with fixed effects represented by Eq. (6) (Wagner & Merlo, 2013, 2015). The lower the PCV, the higher the amount of unexplained variance which can be due to either interaction effects or omitted variables in the model.

References

Evans, C. R., Leckie, G., & Merlo, J. (2020). Multilevel versus single-level regression for the analysis of multilevel information: The case of quantitative intersectional analysis. *Soc Sci Med, 245*, 112499. doi:10.1016/j.socscimed.2019.112499

Fisk, S. A., Mulinari, S., Wemrell, M., Leckie, G., Vicente, R. P., & Merlo, J. (2018). Chronic obstructive pulmonary disease in Sweden: an intersectional multilevel analysis of individual heterogeneity and discriminatory accuracy. *SSM-population health, 4*, 334-346.

Goldstein, H. (2011). *Multilevel statistical models* (Vol. 922): John Wiley & Sons.

Merlo, J. (2018). Multilevel analysis of individual heterogeneity and discriminatory accuracy (MAIHDA) within an intersectional framework. *Soc Sci Med, 203*, 74-80. doi:10.1016/j.socscimed.2017.12.026

Merlo, J., Yang, M., Chaix, B., Lynch, J., & Råstam, L. (2005). A brief conceptual tutorial on multilevel analysis in social epidemiology: investigating contextual phenomena in different groups of people. *Journal of Epidemiology & Community Health, 59*(9), 729-736.

Snijders, T. A., & Bosker, R. J. (2011). *Multilevel analysis: An introduction to basic and advanced multilevel modeling*: sage.

Supplemental file. Retrieved from

Wagner, P., & Merlo, J. (2013). Measures of discriminatory accuracy in multilevel analysis. *Eur J Epidemiol, 28*(1), 135.

Wagner, P., & Merlo, J. (2015). *Discriminatory Accuracy of a Random effect in Multilevel Logistic Regression.* Paper presented at the International Journal of Epidemiology.
