# Supplementary material for: Effects of social determinants on children’s health in informal settlements in Bangladesh and Kenya through an intersectionality lens: a study protocol
Source: BMJ Open. 2022 Jun 6;12(6):e056494. doi: 10.1136/bmjopen-2021-056494 (PMC9171224; doi:10.1136/bmjopen-2021-056494)
Supplement: Author's manuscript [file bmjopen-2021-056494.draft_revisions.pdf]

BMJ Open is committed to open peer review. As part of this commitment we make the peer review history of every article we publish publicly available.

When an article is published we post the peer reviewers' comments and the authors' responses online. We also post the versions of the paper that were used during peer review. These are the versions that the peer review comments apply to.

The versions of the paper that follow are the versions that were submitted during the peer review process. They are not the versions of record or the final published versions. They should not be cited or distributed as the published version of this manuscript.

BMJ Open is an open access journal and the full, final, typeset and author-corrected version of record of the manuscript is available on our site with no access controls, subscription charges or pay-per-view fees (http://bmjopen.bmj.com).

If you have any questions on BMJ Open's open peer review process please email info.bmjopen@bmj.com

BMJ Open

Effects of social determinants on children's health in informal settlements in Bangladesh and Kenya through an intersectionality lens – a study protocol

Journal:	*BMJ Open*
Manuscript ID	bmjopen-2021-056494
Article Type:	Protocol
Date Submitted by the Author:	16-Aug-2021
Complete List of Authors:	Kibuchi, Eliud; University of Glasgow, MRC/CSO Social and Public Health Sciences Unit<br>Barua, Proloy; BRAC, CGSRHR<br>Mithu, Md. Imran Hossain; BRAC, CGSRHR<br>Teixeira de Siqueira Filha, Noemia ; University of York,<br>Phillips-Howard, Penelope; Liverpool School of Tropical Medicine, Department of Clinical Sciences<br>Chumo, Ivy; African Population and Health Research Center<br>Kabaria, Caroline ; African Population and Health Research Center<br>Quayyum , Zahidul ; BRAC University James P Grant School of Public Health,<br>Whittaker, Lana; Liverpool School of Tropical Medicine, Department of Clinical Sciences<br>Dean, Laura; Liverpool School of Tropical Medicine, Department of International Public Health<br>Forsyth, Ross; University of Glasgow, MRC/CSO Social & Public Health Sciences Unit<br>Selim, Tasmiah; BRAC University James P Grant School of Public Health<br>Aktar, Bachera; BRAC University James P Grant School of Public Health<br>Sai, Varun; The George Institute for Global Health India<br>Garimella, Sureka; The George Institute for Global Health India,<br>Saidu, Samuel; COMAHS<br>Gandi , Ibrahim; Centre Of Dialogue On Human Settlement And Poverty Alleviation (CODOHSAPA)<br>Josyula, Lakshmi; The George Institute for Global Health<br>Mberu, Blessing; African Population and Health Research Center<br>Elsey, Helen ; University of York,<br>Leyland, Alastair; University of Glasgow, MRC/CSO Social and Public Health Sciences Unit<br>Gray, Linsay; University of Glasgow, MRC/CSO Social and Public Health Sciences Unit
Keywords:	PUBLIC HEALTH, Community child health < PAEDIATRICS, STATISTICS & RESEARCH METHODS

SCHOLARONE™
Manuscripts

Title: Effects of social determinants on children's health in informal settlements in Bangladesh and Kenya through an intersectionality lens – a study protocol

Eliud Kibuchi[1], *Proloy Barua[2], Md Imran Hossain Mithu[2], Noemia Siqueira[3], Penelope Phillips-Howard[4]*, *Ivy Chumo[5], Caroline Kabaria[5], Zahidul Quayyum[1,2], Lana Whittaker[4], Laura Dean[4]*, Ross Forsyth[1]*, Tasmiah Selim[2], Bachera Aktar[2], Varun Sai[6], Surekha Garimella[6], Samuel Saidu[7], Ibrahim Gandi[8], Joysula k Lakshmi[6], Blessing Mberu[5], Helen Elsey[3], Alastair Leyland[1], Linsay Gray[1]*

[1] University of Glasgow, MRC/CSO Social and Public Health Sciences Unit, Glasgow, UK
[2] James P Grant School of Public Health, Brac University, Dhaka, Bangladesh
[3] University of York, Department of Health Sciences, York, UK
[4] Liverpool School of Tropical Medicine, Pembroke Place, Liverpool, L3 5QA, UK
[5] African Population and Health Research Center, Nairobi, Kenya
The George Institute for Global Health, New Delhi, India
[7] COMAHS: University of Sierra Leone, Freetown, Sierra Leone,
[8] Centre Of Dialogue On Human Settlement And Poverty Alleviation (CODOHSAPA), Freetown, Sierra Leone

Corresponding author: Eliud Kibuchi, Eliud.Kibuchi@glasgow.ac.uk, MRC/CSO Social and Public Health Sciences Unit, Institute of Health and Wellbeing, University of Glasgow, Berkeley Square, 99 Berkeley Street, Glasgow, G3 7HR, UK.

No of words: 3,414

For peer review only - http://bmjopen.bmj.com/site/about/guidelines.xhtml

Abstract

Introduction

Several studies have shown that residents of urban informal settlements are usually excluded and marginalised from formal social systems and structures of power leading to disproportionally worse health conditions compared to other urban dwellers. To promote health equity for slum dwellers, requires an understanding of how their lived realities shape inequities especially among under-fives who tend to have a higher mortality compared with non-slum children. In this proposed study, we aim to examine how key social determinant factors combine to affect under-five health conditions, who live in informal settlements in Bangladesh and Kenya through an intersectionality lens.

Methods and analysis

The protocol describes how we will analyse data from the Nairobi Cross-sectional Slum Survey (NCSS) 2012 for Kenya and the Urban Health Survey (UHS) 2013 for Bangladesh. We will apply multilevel analysis of individual heterogeneity and discriminatory accuracy (MAIHDA) which provides a feasible way of measuring multiple interactions in an intersectionality perspective on children's health conditions. The MAIHDA approach will help us to understand how social determinants intersect with individual factors that shape intersecting inequities to create unique positions of vulnerability for children under five in informal settlements.

Implication and dissemination

The findings from this study will contribute action for promoting policies and intervention strategies for health equity among vulnerable under-five living in informal settlements. In addition, it will bridge the gap between intersectionality theory and application of statistical methods in understanding health inequalities in informal settlements. We will disseminate our results alongside the events organized by the Accountability and Responsiveness in Informal Settlements for Equity (ARISE) consortium and international conferences. Manuscripts will be submitted to an open-access international journal. Ethical approval was not required for these studies. Access to the NCSS (2012) has been given by Africa Population and Health Center and UHS (2013) is freely available.

Key words: Informal settlements, under-five, social determinants, health, intersectionality, MAIHDA

For peer review only

Introduction

People in urban informal settlements face disproportionally worse health conditions compared to other urban dwellers (1). Health conditions are shaped by social determinants–the social characteristics in which living takes place (2). Critically, social determinants help shape social hierarchies that in turn determine the distribution of power, prestige, and resources among groups in society (3). Informal settlement dwellers are usually excluded and marginalised from formal social systems and structures of power, which denies them rights to access resources, which leads to health inequities (1, 4). Empowerment of informal settlement dwellers coupled with investments in health systems and infrastructure is required to attain health equity, particularly amongst socially vulnerable groups (5). In turn, this makes empowered informal settlement dwellers accountable because they have increased collective control over the factors that shape their health.

Lack of data that represent the population in informal settlements across cities has been identified as a major hindrance to answering questions critical to the health needs of the slum dwellers (who constitute most of city dwellers). In turn, this has created obstacles to understanding the health inequities in slum areas for the effective urban health programming by local governments and other stakeholders (6, 7). Currently, 22.8% of the world's population live in slums, and over 90% of slum dwellers live in low and middle income Countries (LMICs), including hundreds of millions of children (8, 9). Despite aggregated statistics showing improved mortality and health outcomes in urban areas, studies show that children in slums experience worse health outcomes than other areas of city and rural areas (6, 10-13).This is because informal settlements are known to have poor services including water drainage, lack of piped water, flooding, poor sewerage, and housing challenges such as overcrowding which are risk factors for waterborne and vector-borne diseases (14-16). For example, infants who live in informal settlements without piped water may have been shown to experience up to 4.8 fold higher rates of death from diarrhea (15). Since the health of children, particularly those under five years old, depends greatly on health and wellbeing of their mothers and families and the broad social determinants factors of where they live; it is crucial to understand how these factors intersect to create complex and unique positions of vulnerability for children in slums(14). To fill the knowledge gap and inform such action, we will systematically examine how various social determinant factors intersect with individual factors to affect the health of under-five children living in informal settlements in (LMICs), through an intersectionality lens.

Social determinants of health are defined based on the commission for social determinants of health (CDSH) framework which classifies them in three main groups:1) the socio-political context; 2) structural determinants and socio-economic status; and 3) intermediary determinants (2, 17, 18). Socio-political refers to those factors in society that cannot be directly measured at the individual level, but which exert a powerful influence on patterns of social stratification and thereby influencing people's health (18). Socio-political factors include labour market, education system and political institutions. Structural determinants are those that generate or reinforce stratification in society, and that define individual socio-economic status within hierarchies of power, prestige, and access to resources (2). Aspects of socio-economic status include income, age, education, occupation, gender, sex, race/ethnicity, sexuality, disability, and social class. On the other hand, intermediary social determinants of health are those factors which socio-economic status operates through to shape health outcomes of individuals and are grouped into four main categories. They are: 1) material circumstances (e.g., housing and neighborhood quality, consumption potential, and physical work environment), 2) psychosocial circumstances (i.e., relationships, social support, and coping styles), 3) behavioral/biological circumstances (i.e., nutrition, physical activity, tobacco consumption, alcohol consumption, substance abuse), and 4) health system, particularly access to health care. Any attempts to address health inequalities especially among the vulnerable and marginalised must focus on understanding how these multiple social determinants interact with individual factors to shape health inequalities.

For peer review only - http://bmjopen.bmj.com/site/about/guidelines.xhtml

An intersectionality lens provides a systematic approach to examining every person's health outcome as fundamentally different from those of others, based on their unique positioning within a web of interacting social determinants (18-21). It assumes that various social determinants of health interact and change through time to present unique circumstances for individuals or population groups. Therefore intersectionality allows us to account for the complexity of the real world in understanding how different social determinants influence health inequities through marginalisation and privilege in multiplicative and interactional ways based on the lived realities of different groups, without the need to make prior assumptions regarding the importance of one or multiple social categories (21, 22). This is achieved by using multilevel analysis of individual heterogeneity and discriminatory accuracy (MAIHDA) that aims to identify intersectional effects by identifying social groups from multiple interactions that are discriminated and disadvantaged than would be expected in absence of these interactions (23). Moreover, an intersectional approach enables us to understand the drivers of health inequities in the context of how individual identities interact with social determinants to promote/rectify health inequalities in dynamic ways in among under-five living in informal settlements (20, 21).

The choice to use intersectionality theory to understand health inequalities for under-five in slums is based on the conceptual framework developed by Accountability and Responsiveness in Informal Settlements for Equity (ARISE) consortium (24). ARISE uses intersectionality theory to analyse and address complex, social economic and political systems that shape health and wellbeing among marginalised urban populations. In our context, intersectionality theory enables critical understanding of power that explain how the multiple social processes and individuals' factors interact to shape health conditions for under-five children who live in informal settlements. In turn, this will contribute to a better understanding of the social context of health for under-fives living in informal settlements. The ARISE consortium works with community members in informal settlements, often as co-researchers, to not only conduct research, but to also respond to the health and well-being challenges facing those living in informal settlements. The findings from our secondary data analysis will inform the work of the ARISE teams in shaping actions to improve health in informal settlements in Bangladesh and Kenya. ARISE also works in India and Sierra Leone, however Bangladesh and Kenya were selected as these were the only two ARISE countries where secondary data that included spatial categorisations of informal settlement was freely available. These two countries are also good examples of rapidly urbanising contexts and allow exploration of the different regional contexts of sub–Saharan African and South Asia.

Aim

We explore how social determinants influence under-five children health conditions among dwellers in informal settlements within an intersectionality framework. The findings will inform how individual and social inequities are shaped, and what action can be taken to offset burdens in terms of effective policy and programme development for vulnerable under-five children.

Objective

The primary analytic objective is to systematically examine how various social determinant and individual factors affect health conditions of under-five children in informal settlements within an intersectionality framework.

Data

In the proposed analysis, we consider that health conditions of under-fives living in informal settlements depends on their demographics, head of household/mothers' demographics and socio-structure characteristics (see Figure 1). We are examining age and sex for under-fives since they have been shown to be determinants of childhood morbidity (25). Moreover, under-five health conditions are closely related to the health and wellbeing of mothers and head of households which affects their ability to provide safe places for under-fives to grow and live (14, 25). Finally, social-

For peer review only - http://bmjopen.bmj.com/site/about/guidelines.xhtml

structure characteristics tend to induce higher or lower prevalence of under-five health conditions since they determine the ability of household to adopt preventive strategies at a given time (18, 26). For example, recent urban migration (i.e. within the last year) has been associated low immunisation rates among under 5 children (27), limited access to health care (28, 29).

Figure 1

In Figure 1, the arrows from under-five child demographics, head of household/mother's demographics and socio-structure characteristics to health conditions represent the hypothesised direct association of social determinants on under-five's health conditions. Specifically, we consider the four health conditions for under-five children: 1) fever, 2) cough, 3) diarrhea, 4) acute respiratory infection (ARI) because they are the most prevalent among children living in informal settlements (10, 14, 30). Their higher prevalence is caused by poor characteristics of physical environment such as poor water drainage, inadequate access to safe water, open sewers, and overcrowding (31). In addition, these health conditions are exacerbated by poor hygiene practices, higher levels of malnutrition and lower immunisation coverage among under-five living in informal settlements (32-36). Considering that health priorities and contexts are different across cities and countries, separate analyses and will be done for Bangladesh and Kenya resulting in two distinct publications. These studies will use cross-sectional data from the Nairobi Cross-section Slums Survey (NCSS) 2012 for Kenya and Urban Health Survey 2013 for Bangladesh, as these were the current disaggregated datasets for slum surveys in both Bangladesh and Kenya. Disaggregated datasets were not available in other countries (India and Sierra Leone), where field activities for ARISE project are being implemented. However, the project team assumes that the outcome will inform all country contexts, as informal settlements in country contexts have almost similar characteristics.

Nairobi Cross-section Slums Survey (NCSS) 2012

The NCSS 2012 was collected by the African Population and Health Research Center (APHRC) from all informal settlements in Nairobi between June and November 2012 (6). A two-stage random sampling methodology was used and a total of 5,490 households and 4,420 women aged 12-49 years were successfully interviewed yielding a response rate of 88 and 86 percent respectively. We are interested in women because their questionnaire contained a module on child's health, where we are to obtain our health outcomes of interest. A total of 2,199 children's data aged under 5 years was provided in the women's questionnaire.

In this study we consider three health conditions for children: 1) whether a child had fever or not, 2) whether a child had cough or not, and 3) whether a child had diarrhea or not. ARI is not available in the (NCSS) 2012 and will therefore not be considered. The predictor variables to be considered in the analysis are classified into three categories: 1) children's demographics (i.e., age and sex), head of household characteristics (i.e., sex, ethnic group, and age group) and 3) social structure characteristics in the household (i.e., wealth quintile, length of stay, religion, education, tenure, food availability, health insurance, income generating activity and catastrophic health costs at 40% threshold). The wealth quintile index was generated using source of drinking water, type of toilet facility, cooking fuel used, lighting type at night, material used to construct floor, wall and roof of dwelling, and household possessions (ownership of household items) (6). Catastrophic health expenditure was computed using the empirical methodological procedure used by (37) and 40% threshold was informed by (38) (see Figure 2).

Figure 2

For peer review only - http://bmjopen.bmj.com/site/about/guidelines.xhtml

Bangladesh Urban Health Survey (UHS) 2013 data

The Urban Health Survey (UHS) 2013 is a representative cross-sectional household survey implemented jointly by National Institute of Population Research and Training (NIPORT), Measure Evaluation, University of North Carolina at Chapel Hill, USA, icddr,b and Associates for Community and Population Research, a Bangladeshi private research agency (39). The survey collected information designed to examine intra-urban differentials in heath and service utilisation from 53,790 households. These households were selected using a stratified three-stage sampling procedure in three urban domains: 1) City corporation slum, 2) City corporation non-slum, and 3) other urban areas. The proposed analysis will only include the domain of city corporation slum since our interest involves investigating social processes which drive health inequalities in informal settlements. In addition, we will consider the women subsample from the Survey because their questionnaire contained a module on child's health and nutrition. The number of households selected in the domain of city corporation slum were 15,750 and those interviewed 14,806 yielding a response rate of 94 percent. A total of 14,702 women were eligible for interview and 14,011 were interviewed yielding a 95% response rate.

We considered three child health conditions: 1) whether a child had fever or not, 2) whether a child had cough or not, and 3) whether a child had an acute respiratory infection (ARI) or not. ARI is a cough accompanied by short, rapid, or difficult breathing which is chest related and usually considered as a proxy for pneumonia. Diarrhea will not be considered because it is not available in the (UHS) 2013.The predictor variables to be considered in the analyses are classified into four categories: 1) children's demographics (i.e., age and sex), 2) mother's demographics (i.e., religion, age, highest level of education, employment status), 3) head of household demographics (i.e., sex, and age) and 4) social structure characteristics in the household (i.e., wealth quintile, fuel used for cooking, garbage disposal method and length of stay) (see Figure 3). Wealth quintile index was constructed using principal components analysis (PCA) based on the following variables: dwelling characteristics such as presence of electricity, type of water source, type of toilet, and floor, wall, and roof material, household ownership of selected assets and durable goods (radio, television, motorcycle, computer, refrigerator, electric fan, and automobile), and two indicators of housing tenure (whether the household held title to the dwelling and/or the land).

Figure 3

Statistical methods

The effects of social determinants on children's health outcomes in informal settlements through an intersectionality lens will be assessed using inter and intra categorical analysis technique known as multilevel analysis of individual heterogeneity and discriminatory accuracy (MAIHDA) (19-21). MAIHDA aims to primarily identify intersectional effects by identifying groups that are advantaged than would be expected in the absence of interaction by distinguishing between additive and interaction effects (40, 41). This allows consideration of both group averages and multilevel variation within and between groups by decomposing the mean outcome for any intersectional group into the additive effects of attribute at each intersection, and additional intersectional effect specific to that group. MAIHDA therefore, allows multiplicative modelling of health inequalities at the intersection of multiple social determinants by analysing the heterogeneity (i.e. differences) within and between intersectional social determinants groups by separating variance (i.e. the measure of variation) into – the between-strata (i.e. between category) and the within - strata (i.e. within category) variance

For peer review only - http://bmjopen.bmj.com/site/about/guidelines.xhtml

(40). MAIHDA also allows quantification of discriminatory accuracy (DA) of the intersectional group by discerning those who have an outcome of interest from those who don't using Variance Partitioning Coefficient (VPC) and the area under the receiver operating characteristic curve (AUC) (40-42). VPC represents the proportion of the total individual variance in the outcome of interest that is accounted for at the intersectional level (43, 44). On the other hand, AUC measures the ability of the model to classify individuals with or without outcome of interest as a function of individual's predicted probabilities (43, 44).

The advantage of MAIHDA that we can look at intersectionality as a mix of both marginalisation and privilege (40, 42, 45). Generally, an interaction-based approach looks at intersectionality from the perspective of marginalisation only, which runs the risk of reinforcing the notion of social dominance of the privileged groups which are used as "default" categories. In addition, from an analytical perspective, multilevel models do not face the issues of scalability (i.e., a model's inability to accommodate an increase in the number of variables included), model parsimony (i.e., a simple model not having great explanatory predictive power), and reduced sample size in some intersectional groups (which influences whether an effect size is determined or not) (41, 46). If desired, we can extend this multilevel framework into a multivariate multilevel model to analyse more than two health outcomes simultaneously, especially for health conditions which occur concurrently affecting other aspects of life (47, 48).

Multilevel models which are mainly used when modelling clustering of individuals based on some shared attributes such as neighborhood, school, or household, among others. The adoption of multilevel models is therefore motivated by the fact that clustering of individuals can also include abstract groupings such as a set of social determinants or processes associated with their intersectional social identities and individual characteristics. As an illustrative example, consider investigating the effects of child's age (i.e., up to 1 year, 2 to 3 years., 4 to 5 years), child's sex (i.e., female or male), head of household education (none, primary, secondary and higher), sex of the head of household (i.e., male or female), age of the head of household (i.e., 18-25 years, 26-32 years, 33-40 years, >40 years) and household covered by health insurance (yes or no). Using the six variables and their corresponding categories: child's age (3), child's sex (2), head of household education (3), head of household sex (2), head of household age (4) and household health insurance (2) we can create 288 groups/strata. The first stratum can consist of a child who is a female, aged up to 1 year and less, coming from a household whose head is a female, aged 18-25 years and with no education and the household is not covered by health insurance, and the process continues until all the children at level 1 are nested within the groups/strata ($N = 288$) at level 2. This means that children at level 1 who share similar categories of social processes end up being in the same group/stratum at level 2.

To capture differences in disparities in children health conditions between different groups, for example - males and females, we can assess child's sex-specific effects through their interactions with child 's age, head of household's sex, age and education and household's health insurance using fixed effects model. However, fixed effects model formulation only addresses interactions between child's sex and each of the other five variables. If we were to consider all possible interactions among the six variables, we will have 288 interaction terms in the regression model which may result in the model having parsimony and scalability issues due to geometrical growth of coefficients as more variables are included in the model. In addition, it would be difficult to interpret the results and reduced sample size in some interaction groups may influence whether an effect size is determined or not (41, 46, 48). However, we can overcome these limitations of fixed effects models by applying MAIHDA approach (41), by treating social strata/groups defined by child's age and sex, head of household sex, age and education and household's health insurance as clusters (Appendix Eq. 6).

For peer review only - http://bmjopen.bmj.com/site/about/guidelines.xhtml

In MAIHDA approach, we capture the unique interaction effect for each social group/stratum (i.e., social strata -specific differences in child's diarrhea) while accounting for sample size differences for each social stratum/group (40, 41). This implies that MAIHDA provides a feasible way of measuring multiple interactions and analysing groups of small size in an intersectionality perspective. This improves understanding of diverse distribution and determinants of individual health in the population and social processes that drive health inequalities.

The model can even be extended to include more than two health conditions in a multivariate multilevel model (47, 48). This model explicitly evaluates the covariance (i.e., a measure of joint variability of two random variables) between different social strata and health conditions which allows us not only to draw conclusions about social group-specific differences but also correlations between health conditions (Appendix Eq. 9). This is desirable since we assume that they capture related, though distinct, health constructs.

Limitation of this proposed study is that datasets which will be used were collected over eight years ago (6, 39). Considering the dynamic nature of informal settlements, a more recent data would have been more informative of the social processes which affect under-five health conditions. Despite this, we expect the findings obtained to be of great value since these datasets come from the most recently conducted slum surveys in both Bangladesh and Kenya.

Conclusion

To conclude, the proposed analysis framework will allow investigation of effects of social determinants on under-five health conditions in informal settlements through an intersectionality lens. The exploration of these determinants in the two different country contexts will provide insights on differences and similarities dependent on context and extent of urban transition. Knowledge gained on how social determinants interact to impact on multiple aspects of health in under-fives in informal settlements will ultimately contribute to the overall aim of ARISE by informing action for promoting policies and intervention strategies for health equity for vulnerable under-five living in informal settlements. Moreover, our proposed approach will bridge the gap between intersectionality theory and application of statistical methods in understanding health inequalities in informal settlements.

Funding

The GCRF Accountability for Informal Urban Equity Hub ('ARISE') is a UKRI Collective Fund award with award reference ES/S00811X/1. The Social and Public Health Sciences Unit is funded by the Medical Research Council (MC_UU_00022/2) and the Scottish Government Chief Scientist Office (SPHSU17).

Author's contribution

EK prepared the first draft of this paper. EK, LG and AL were involved in the conception of the study design, literature search and contributed all sections of the paper. PB, IHM, NS, PPH, IC, CK, ZD, LW, LD, RF, TS, BA, VS, SG, SS, IG, JLK, BM and HE contributed to all sections and the literature search. PB, IHM, ZD, IC, CK, BM, PM facilitated the acquisition of the survey data. All authors contributed to all sections of the manuscript and approved the final version

For peer review only - http://bmjopen.bmj.com/site/about/guidelines.xhtml

For peer review only

For peer review only - http://bmjopen.bmj.com/site/about/guidelines.xhtml

References

1. World Health Organization. A billion voices: listening and responding to the health needs of slum dwellers and informal settlers in new urban settings. World Health Organization; 2005.
2. World Health Organization. A conceptual framework for action on the social determinants of health. 2010.
3. Tarlov AR. Social determinants of health: the sociobiological translation. Health and social organization: Routledge; 2002. p. 87-109.
4. Dovey K, King R. Forms of informality: Morphology and visibility of informal settlements. Built Environment. 2011;37(1):11-29.
5. Organization WH. Our cities, our health, our future: acting on social determinants for health equity in urban settings. Report to the WHO Commission on Social Determinants of Health from the Knowledge Network on Urban Settings. Our cities, our health, our future: acting on social determinants for health equity in urban settings Report to the WHO Commission on Social Determinants of Health from the Knowledge Network on Urban Settings. 2008.
6. APHRC. Population and health dynamics in Nairobi's informal settlements: Report of the Nairobi Cross-sectional Slums Survey (NCSS) 2012. Nairobi: APHRC. 2014(April):1-185.
7. Satterthwaite D, editor Health in urban slums depends on better local data. International Conference on Urban Health, Manchester, UK; 2014.
8. UNICEF. Children in an Urban World: the State of the World's Children 2012. Retrieved July. 2012;24:2012.
9. Economic UNDo, Division SAP. World urbanization prospects: The 2018 revision: UN; 2019.
10. Kyobutungi C, Ziraba AK, Ezeh A, Yé Y. The burden of disease profile of residents of Nairobi's slums: Results from a Demographic Surveillance System. Population health metrics. 2008;6(1):1-8.
11. Barrett JR. A marked disadvantage: rapid urbanization and mortality of young children in Nigeria. National Institute of Environmental Health Sciences; 2010.
12. APHRC. Population and health dynamics in Nairobi's informal settlements: report of the Nairobi Cross-sectional Slums Survey (NCSS) 2000: African Population and Health Research Center; 2002.
13. UNICEF. Understanding urban inequalities in Bangladesh: a prerequisite for achieving Vision 2021. UNICEF Bangladesh, Dhaka. 2010.
14. Unger A. Children's health in slum settings. Arch Dis Child. 2013;98(10):799-805.
15. Victora CG, Smith PG, Vaughan JP, Nobre LC, Lombard C, Teixeira AMB, et al. Water supply, sanitation and housing in relation to the risk of infant mortality from diarrhoea. International journal of epidemiology. 1988;17(3):651-4.
16. World Health Organization. Hidden cities: unmasking and overcoming health inequities in urban settings: World Health Organization. Centre for Health Development; 2010.
17. Commission on Social Determinants of Health. Closing the gap in a generation: health equity through action on the social determinants of health: final report of the commission on social determinants of health: World Health Organization; 2008.
18. Organization WH. A conceptual framework for action on the social determinants of health. 2010.
19. Green MA, Evans CR, Subramanian SV. Can intersectionality theory enrich population health research? 2017.
20. López N, Gadsden VL. Health inequities, social determinants, and intersectionality. 2016.
21. Sen G, Iyer A. Who gains, who loses and how: leveraging gender and class intersections to secure health entitlements. Social science & medicine. 2012;74(11):1802-11.
22. Larson E, George A, Morgan R, Poteat T. 10 Best resources on… intersectionality with an emphasis on low-and middle-income countries. Health policy and planning. 2016;31(8):964-9.
23. . !!! INVALID CITATION !!! (11, 12).

For peer review only - http://bmjopen.bmj.com/site/about/guidelines.xhtml

24. ARISE. Accountability in Urban Health 2021 [updated 27 July 2021. Available from: https://www.ariseconsortium.org/.
25. Taffa N, Chepngeno G, Amuyunzu-Nyamongo M. Child morbidity and healthcare utilization in the slums of Nairobi, Kenya. Journal of Tropical Pediatrics. 2005;51(5):279-84.
26. Ensor T, Bhattarai R, Manandhar S, Poudel AN, Dhungel R, Baral S, et al. From Rags to Riches: Assessing poverty and vulnerability in urban Nepal. PloS one. 2020;15(2):e0226646.
27. Crocker-Buque T, Mindra G, Duncan R, Mounier-Jack S. Immunization, urbanization and slums–a systematic review of factors and interventions. BMC public health. 2017;17(1):1-16.
28. Adhikary P, Aryal N, Dhungana RR, Kc RK, Regmi PR, Wickramage KP, et al. Accessing health services in India: experiences of seasonal migrants returning to Nepal. BMC health services research. 2020;20(1):1-7.
29. Afeadie RK. Rural–urban drift: labour migration, health-seeking behaviour disparity in the urban slum of Madina, Ghana. Health Education. 2021.
30. Hussain A, Ali SK, Kvåle G. Determinants of mortality among children in the urban slums of Dhaka city, Bangladesh. Tropical Medicine & International Health. 1999;4(11):758-64.
31. UN-HABITAT. The challenge of slums: global report on human settlements, 2003: UN-HABITAT -United Nations Human Settlements Programme Staff; 2003.
32. Fotso J-C. Child health inequities in developing countries: differences across urban and rural areas. International journal for equity in health. 2006;5(1):1-10.
33. Fry S, Cousins B, Olivola K. Health of children living in urban slums in Asia and the near east: Review of existing literature and data. Washington, DC: Environmental Health Project, US Agency for International Development. 2002.
34. Sverdlik A. Ill-health and poverty: a literature review on health in informal settlements. Environment and urbanization. 2011;23(1):123-55.
35. Butala NM, VanRooyen MJ, Patel RB. Improved health outcomes in urban slums through infrastructure upgrading. Social science & medicine. 2010;71(5):935-40.
36. Agarwal S, Bhanot A, Goindi G. Understanding and addressing childhood immunization coverage in urban slums. Indian pediatrics. 2005;42(7):653.
37. Buigut S, Ettarh R, Amendah DD. Catastrophic health expenditure and its determinants in Kenya slum communities. International journal for equity in health. 2015;14(1):1-12.
38. Xu K, Aguilar A, Carrin G, Evans D, Hanvoravongchai P, Kawabata K, et al. Distribution of health payments and catastrophic expenditures methodology. 2005. World Health Organization, Discussion Paper. 2017(2).
39. NIPORT ICfDDR, Bangladesh ,, Evaluation M. Bangladesh urban health survey 2013. Final report. NIPORT, ICDDRB, and MEASURE Evaluation Dhaka, Bangladesh and Chapel Hill …; 2015.
40. Evans CR, Williams DR, Onnela JP, Subramanian SV. A multilevel approach to modeling health inequalities at the intersection of multiple social identities. Soc Sci Med. 2018;203:64-73.
41. Merlo J. Multilevel analysis of individual heterogeneity and discriminatory accuracy (MAIHDA) within an intersectional framework. Soc Sci Med. 2018;203:74-80.
42. Evans CR, Leckie G, Merlo J. Multilevel versus single-level regression for the analysis of multilevel information: The case of quantitative intersectional analysis. Soc Sci Med. 2020;245:112499.
43. Wagner P, Merlo J. Measures of discriminatory accuracy in multilevel analysis. Eur J Epidemiol. 2013;28(1):135.
44. Merlo J, Wagner P, Ghith N, Leckie G. An original stepwise multilevel logistic regression analysis of discriminatory accuracy: the case of neighbourhoods and health. PloS one. 2016;11(4):e0153778.
45. Fisk SA, Mulinari S, Wemrell M, Leckie G, Vicente RP, Merlo J. Chronic obstructive pulmonary disease in Sweden: an intersectional multilevel analysis of individual heterogeneity and discriminatory accuracy. SSM-population health. 2018;4:334-46.
46. Goldstein H. Multilevel statistical models: John Wiley & Sons; 2011.

For peer review only - http://bmjopen.bmj.com/site/about/guidelines.xhtml

47. Baldwin SA, Imel ZE, Braithwaite SR, Atkins DC. Analyzing multiple outcomes in clinical research using multivariate multilevel models. Journal of consulting and clinical psychology. 2014;82(5):920.
48. Snijders TA, Bosker RJ. Multilevel analysis: An introduction to basic and advanced multilevel modeling: sage; 2011.

For peer review only

For peer review only - http://bmjopen.bmj.com/site/about/guidelines.xhtml

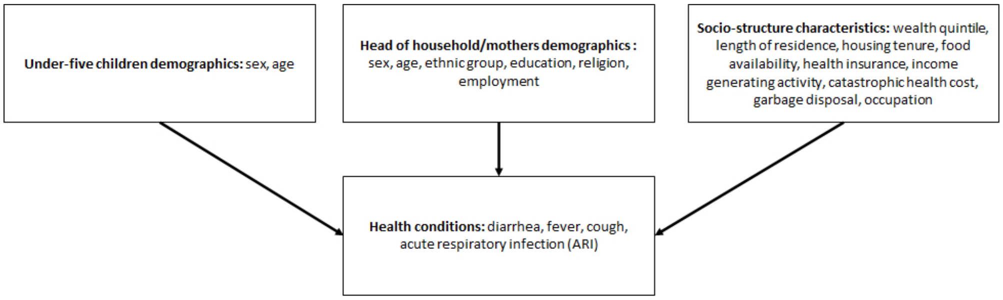

Figure 1: Flow diagram to understand associations between under-five demographics, head of household/mothers' demographics, socio-structure characteristics, and under-five health conditions.

652x229mm (38 x 38 DPI)

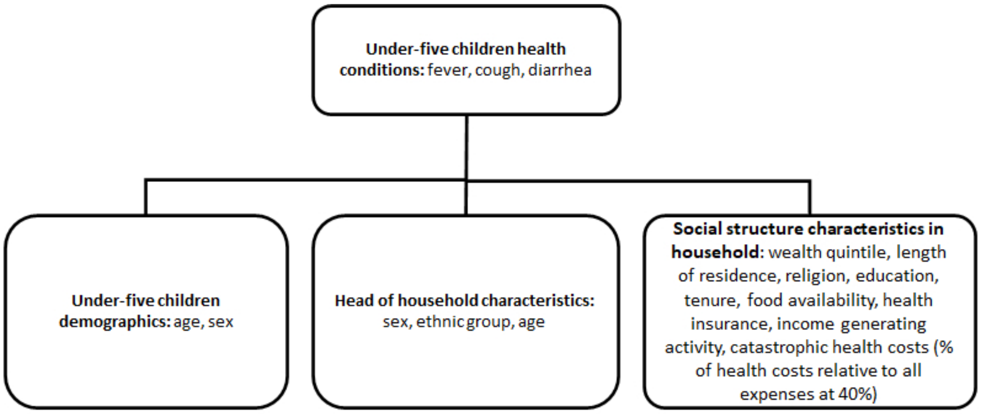

Figure 2: Children health conditions sociodemographic characteristics in the Nairobi Cross-section slums survey (NCSS) 2012.

542x227mm (38 x 38 DPI)

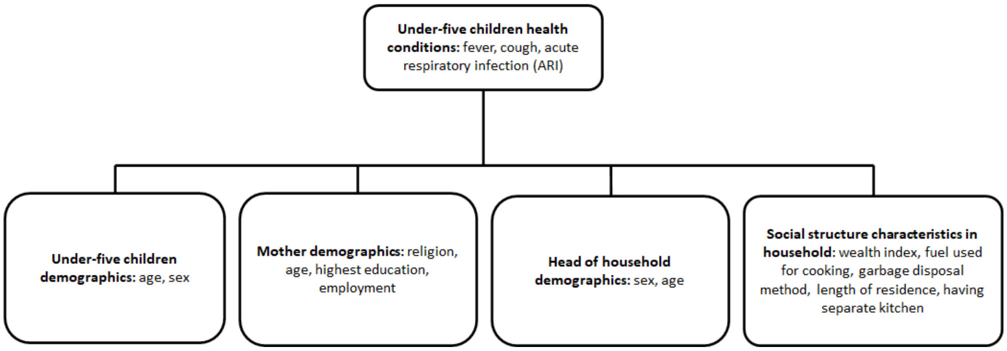

Figure 3: Children health conditions and sociodemographic characteristics in the Bangladesh Urban Health Survey (UHS) 2013

697x239mm (38 x 38 DPI)

Supporting information: Statistical methodology details

Let us consider the case where we are interested in investigating effects of child's age (i.e., up to 1 year and less, 2 to 3 years., 4 to5 years), child's sex (i.e., female or male), head of household sex (i.e., male or female), head of household education (none, primary, secondary and higher), head of household age (i.e.,18-25 years, 26-32 years, 33-40 years., >40 years) and household health insurance (yes or no) on children's diarrhea in slums.
Let y_i denote a binary health outcome (i.e., diarrhea) for child $i(i = 1,\ldots,i)$ where,

$$y_i = \begin{cases} 0 \;\; absence\ of\ diarhea \\ 1 \;\; Presence\ of\ diarrhea \end{cases} \quad \text{Eq.(1)}$$

y_i is assumed to follow a Bernoulli distribution, with probabilities $\pi_i = Pr(y_i = 0)$ the probability of child i having no diarrhea and $1 - \pi_i = Pr(y_i = 1)$ the probability of child i having diarrhea. Let X_{1i} represent child sex, X_{2i} represent child's age, X_{3i}represent head of household sex, X_{4i} represent head of household education, X_{5i} represent head of household age and X_{6i} represent household health insurance. These six variables represent our explanatory variables. Logistic regression is appropriate for modelling binary (two category) outcomes such as whether a child has diarrhea or not.

The fixed effects logistic regression model for investigating how child sex, child age, head of household sex, head of household education, head of household age and household health insurance are additively associated with child's diarrhea is represented in equation 1 "Eq. (2)".

$$log\left(\frac{\pi_i}{1-\pi_i}\right) = \beta_0 + \beta_1 X_{1i} + \beta_2 X_{21i} + \beta_3 X_{22i} + \beta_4 X_{3i} + \beta_5 X_{41i} + \beta_6 X_{42i} + \beta_7 X_{51i} + \beta_8 X_{52i} + \beta_9 X_{53i} + \beta_{10} X_{6i} \quad \text{Eq. (2)}$$

Eq. (2) estimates the associations of child sex, child age, head of household sex, head of household education, head of household age and household health insurance with child's diarrhea additively (i.e., explanatory effects) and does not accommodate for interactions with each other. In order, to capture specific effects between different groups, for example, child's sex (i.e., males or females), we can assess sex-specific disparities in diarrhea through their interactions with child age, head of household sex, head of household education, head of household age and household health insurance.

Eq. (2) can thus be expanded to include interaction terms, as presented as follows in Eq. (3):

$$log\left(\frac{\pi_i}{1-\pi_i}\right) = \beta_0 + \beta_1 X_{1i} + \beta_2 X_{21i} + \beta_3 X_{22i} + \beta_4 X_{3i} + \beta_5 X_{41i} + \beta_6 X_{42i} + \beta_7 X_{51i} + \beta_8 X_{52i} + \beta_9 X_{53i} + \beta_{10} X_{6i} + \beta_{11} X_{21i} X_{1i} + \beta_{12} X_{22i} X_{1i} + \beta_{13} X_{3i} X_{1i} + \beta_{14} X_{41i} X_{1i} + \beta_{15} X_{42i} X_{1i} + \beta_{16} X_{51i} X_{1i} + \beta_{17} X_{52i} X_{1i} + \beta_{18} X_{53i} X_{1i} + \beta_{19} X_{6i} X_{1i} \quad \text{Eq. (3)}$$

whereβ_{11} to β_{19} are interaction coefficients between child's sex and other explanatory variables in Eq. (2). Not only does Eq. (3) allow for an analysis that considers the association of child's sex in getting diarrhea but also uncovers how other factors that create and sustain diarrhea may differ based on the sex of child. However, Eq. (3) only addresses interactions between child's sex and the other five variables and if we were to consider all possible interactions among the six variables, we will have a total of 288 fixed effects in the logistic model. The higher number of fixed effects may lead to issues with scalability (i.e., a model's inability to accommodate an increase in the number of variables included), model parsimony (i.e., a simple model not having great explanatory predictive power) and reduced sample size in some intersectional groups which may influence whether an effect is determined is determined or not. In addition, it would be difficult to interpret 288 fixed

For peer review only - http://bmjopen.bmj.com/site/about/guidelines.xhtml

effects. We can overcome these issues by using multilevel analysis of individual heterogeneity and discriminatory accuracy (MAIHDA) approach.

Multilevel analysis of individual heterogeneity and discriminatory accuracy (MAIHDA)

Now, let us consider the case where we are interested in investigating effects child's age (i.e., up to 1 year and less, 2 to 3 years., 4 to5 years), child's sex (i.e., female or male), head of household sex (i.e., male or female), head of household education (none, primary, secondary and higher), head of household age (i.e.,18-25years, 26-32years, 33-40 years, >40years) and household health insurance (yes or no) on the outcome diarrhea using MAIHDA. The first step in this approach involves creating groups/strata based on the categories of the social determinants factors we are interested in. This means that children at level 1 who share similar categories of social determinants factors will end up being in the same group/strata at level 2. Therefore, we will have individuals at level 1 nested within 288 groups at level 2.

Therefore, let y_{ij} denote a binary health outcome (i.e., diarrhea) for child $i(i = 1,\ldots,n)$ in groups j $(j = 1,\ldots,N)$ where,

$$y_{ij} = \begin{cases} 0 & absence\ of\ diarrhea \\ 1 & Presence\ of\ diarrhea \end{cases} \quad \text{Eq.(4)}$$

y_{ij} is assumed to follow a Bernoulli distribution, with probabilities $\pi_{ij} = Pr(y_{ij} = 0)$ the probability of child i from group j having no diarrhea and $1 - \pi_{ij} = Pr(y_i = 1)$ the probability of child i from group j having diarrhea. Let X_{1ij} represent child sex, X_{2ij} represent child's age, $X_{3ij}j$represent head of household sex, X_{4ij} represent head of household education, X_{5ij} represent head of household age and X_{6ij} represent household health insurance.

The fixed effects logistic regression model for investigating how child sex, child age, head of household sex, head of household education, head of household age and household health insurance are additively associated with child's diarrhea are additively associated with diarrhea can be extended to incorporate groups/strata as represented in Eq. (6)

$$log\left(\frac{\pi_{ij}}{1-\pi_{ij}}\right) = \beta_0 + \beta_1 X_{1ij} + \beta_2 X_{21ij} + \beta_3 X_{22ij} + \beta_4 X_{3ij} + \beta_5 X_{41ij} + \beta_6 X_{42ij} + \beta_7 X_{51ij} + \beta_8 X_{52ij} + \beta_9 X_{53ij} + \beta_{10} X_{6ij} \quad \text{Eq. (5)}$$

Equation 5 can be extended by taking the MAIHDA approach {Merlo, 2018 #375} by treating social groups defined by child sex, child age, head of household sex, head of household education, head of household age and household health insurance as clusters which are associated multiplicatively with a child developing diarrhea or not. Therefore Eq. (5) can be extended to:

$$log\left(\frac{\pi_{ij}}{1-\pi_{ij}}\right) = \beta_0 + \beta_1 X_{1ij} + \beta_2 X_{21ij} + \beta_3 X_{22ij} + \beta_4 X_{3ij} + \beta_5 X_{41ij} + \beta_6 X_{42ij} + \beta_7 X_{51ij} + \beta_8 X_{52ij} + \beta_9 X_{53ij} + \beta_{10} X_{6ij} + \mu_{0j} \quad \text{Eq. (6)}$$

where β_0 is the intercept and $\mu_{0j} \sim N(0, \sigma^2_{group})$ represents the group level residual which is normally distributed with mean 0 and variance σ^2_{group}. Assuming no omitted variable bias, the group level residual μ_{0j} captures the unique interaction effect for each social group/strata (i.e. social groups - specific differences in diarrhea) while accounting for sample size differences for each social group. The relevance of the intersectional strata for understanding individual heterogeneity is evaluated using Variance partitioning Coefficient (VPC) also known as intraclass coefficient (which also informs on the discriminatory accuracy of the intersectional categorisation for distinguishing children with diarrhea from those without.

For peer review only - http://bmjopen.bmj.com/site/about/guidelines.xhtml
For peer review only

$$ICC = \left(\frac{\sigma^2_{group}}{\sigma^2_{group} + 3.29}\right) \text{ Eq. (7)}$$

where the value 3.29 is the variance for the standard logistic distribution (1).

If desirable, this model can be further extended to include more than two health outcomes in a multivariate multilevel model (2). For example, let's assume that respondents (level 2), social group (level 3) j, with h health outcome variables, $y_1, \ldots, y_m$ (i.e., $h = 1, \ldots, m$)at level 1 and x_{kij} explanatory variables (i.e., $k = 1, \ldots, p$). The outcome variable is denoted as y_{hij} which is the outcome on the h'th health variable for respondent i in social group j. Another useful feature of multivariate multilevel model is that we can account for missing health outcomes not recorded for some respondents by assume that missingness is due to missing at random (MAR) (2). That is, conditionally given the observed data, the missingness indicators are independent of the unobserved data (3).

We can define the complete data vector for the m health outcomes by combining them into a single outcome variable y_{hij}as

$$y_{hij} = (y_{1ij} \ldots y_{mij}) \quad \text{Eq. (8)}$$

In addition, we also create dummy variables $d_1, \ldots, d_m$ which are defined for $h = 1, \ldots, m$,

$$d_{shij} = \{1(h = s)0(h \neq s) \quad \text{Eq. (9)}$$

where dummy variable d_h is 1 or 0, depending on whether the data line refers to outcome variable y_h or to one of the other health outcome variables. With these dummies, the random intercept model Eq. (6) for the m health outcomes can be defined as

$$y_{hij} = \sum_{s=1}^{m} \beta_{0s} d_{shij} + \sum_{k=1}^{p} \sum_{s=1}^{m} \beta_{ks} d_{shij} x'_{kij} + \sum_{s=1}^{m} \mu_{0sj} d_{shij} \text{ Eq. (10)}$$

where all variables including the constant are multiplied by the dummy variables d_{shij}. $\beta_{0s}$is the intercept, $x'_{kij}$is a vector of social determinants variables with coefficient vector β while μ_{0j} is a group level residual which follow a multivariate normal distribution $\Sigma = cov(\mu_{0j})$. Therefore, Eq. (10) represent the multivariate data in a multilevel approach with level 1 being health outcomes variables indexed by $h = 1, \ldots, m$, level 2 represents respondents $i = 1, \ldots, n_j$, and level 3 represents social group defined by social determinants variables. This set up models explicitly the covariance between different social groups and health outcomes which allows us not only to draw conclusions about social group-specific differences but also correlations between health outcomes are desirable since we assume that they capture related, though distinct, health constructs.

References

1. Goldstein H. Multilevel statistical models: John Wiley & Sons; 2011.
2. Snijders TA, Bosker RJ. Multilevel analysis: An introduction to basic and advanced multilevel modeling: sage; 2011.
3. Rubin; DB. Inference and Missing Data. Biometrika. 1976;63(3):581-92.

For peer review only

BMJ Open

Effects of social determinants on children's health in informal settlements in Bangladesh and Kenya through an intersectionality lens – a study protocol

Journal:	*BMJ Open*
Manuscript ID	bmjopen-2021-056494.R1
Article Type:	Protocol
Date Submitted by the Author:	14-Mar-2022
Complete List of Authors:	Kibuchi, Eliud; University of Glasgow, MRC/CSO Social and Public Health Sciences Unit<br>Barua, Proloy; BRAC, CGSRHR<br>Chumo, Ivy; African Population and Health Research Center<br>Teixeira de Siqueira Filha, Noemia ; University of York,<br>Phillips-Howard, Penelope; Liverpool School of Tropical Medicine, Department of Clinical Sciences<br>Mithu, Md. Imran Hossain; BRAC, CGSRHR<br>Kabaria, Caroline ; African Population and Health Research Center<br>Quayyum , Zahidul ; BRAC University James P Grant School of Public Health,<br>Whittaker, Lana; Liverpool School of Tropical Medicine, Department of Clinical Sciences<br>Dean, Laura; Liverpool School of Tropical Medicine, Department of International Public Health<br>Forsyth, Ross; University of Glasgow, MRC/CSO Social & Public Health Sciences Unit<br>Selim, Tasmiah; BRAC University James P Grant School of Public Health<br>Aktar, Bachera; BRAC University James P Grant School of Public Health<br>Sai, Varun; The George Institute for Global Health India<br>Garimella, Sureka; The George Institute for Global Health India,<br>Saidu, Samuel; COMAHS<br>Gandi , Ibrahim; Centre Of Dialogue On Human Settlement And Poverty Alleviation (CODOHSAPA)<br>Josyula, Lakshmi; The George Institute for Global Health<br>Mberu, Blessing; African Population and Health Research Center<br>Elsey, Helen ; University of York,<br>Leyland, Alastair; University of Glasgow, MRC/CSO Social and Public Health Sciences Unit<br>Gray, Linsay; University of Glasgow, MRC/CSO Social and Public Health Sciences Unit
**Primary Subject Heading**:	Epidemiology
Secondary Subject Heading:	Global health, Public health
Keywords:	PUBLIC HEALTH, Community child health < PAEDIATRICS, STATISTICS & RESEARCH METHODS, EPIDEMIOLOGY

SCHOLARONE™
Manuscripts

Effects of social determinants on children's health in informal settlements in Bangladesh and Kenya through an intersectionality lens – a study protocol

Eliud Kibuchi[1], Proloy Barua[2], Ivy Chumo[3], Noemia Siqueira[4], Penelope Phillips-Howard[5], Md Imran Hossain Mithu[2], Caroline Kabaria[3], Zahidul Quayyum[1,2], Lana Whittaker[5], Laura Dean[5], Ross Forsyth[1], *Tasmiah Selim[2], Bachera Aktar[2], Varun Sai[6], Surekha Garimella[6], Samuel Saidu[7], Ibrahim Gandi[8], Joysula k Lakshmi[6], Blessing Mberu[3], Helen Elsey[4], Alastair Leyland[1], Linsay Gray[1]*

[1] University of Glasgow, Glasgow, UK
[2] James P Grant School of Public Health, Brac University, Dhaka, Bangladesh
[3] African Population and Health Research Center, Nairobi, Kenya
[4] University of York, Department of Health Sciences, York, UK
[5] Liverpool School of Tropical Medicine, Pembroke Place, Liverpool, L3 5QA, UK
[6]The George Institute for Global Health, New Delhi, India
[7] COMAHS: University of Sierra Leone, Freetown, Sierra Leone,
[8] Centre Of Dialogue On Human Settlement And Poverty Alleviation (CODOHSAPA), Freetown, Sierra Leone

Corresponding author: Eliud Kibuchi, Eliud.Kibuchi@glasgow.ac.uk, MRC/CSO Social and Public Health Sciences Unit, Institute of Health and Wellbeing, University of Glasgow, Berkeley Square, 99 Berkeley Street, Glasgow, G3 7HR, UK.

Abstract no of words: 283

No of words: 3,906

For peer review only - http://bmjopen.bmj.com/site/about/guidelines.xhtml

Abstract

Introduction

Several studies have shown that residents of urban informal settlements/slums are usually excluded and marginalised from formal social systems and structures of power leading to disproportionally worse health conditions compared to other urban dwellers. To promote health equity for slum dwellers, requires an understanding of how their lived realities shape inequities especially for young children 0-4 years old (i.e., under-fives) who tend to have a higher mortality compared with non-slum children. In these proposed studies, we aim to examine how key Social Determinants of Health (SDoH) factors at child and household levels combine to affect under-five health conditions, who live in slums in Bangladesh and Kenya through an intersectionality lens.

Methods and analysis

The protocol describes how we will analyse data from the Nairobi Cross-sectional Slum Survey (NCSS 2012) for Kenya and the Urban Health Survey (UHS 2013) for Bangladesh. The analysis sample will be based on complete case analyses with variables to be included in the analyses will be selected via univariate analyses. We will apply Multilevel Analysis of Individual Heterogeneity and Discriminatory Accuracy (MAIHDA) to explore multiple interactions in an intersectionality perspective on children's health conditions. Some of SDoH characteristics to be considered will include child's age and sex, head of household characteristics such as sex and age and social structure characteristics of household such as wealth index, residence status among others. The primary outcome measures will be diarrhea, cough, fever, and acute respiratory infection (ARI).

Ethics and dissemination

The results will be disseminated in international peer-reviewed journals and presented in events organized by the Accountability and Responsiveness in Informal Settlements for Equity (ARISE) consortium and international conferences. Ethical approval was not required for these studies. Access to the NCSS 2012 has been given by Africa Population and Health Center and UHS 2013 is freely available

Key words: Informal settlements/slums, under-five, social determinants of health, intersectionality, MAIHDA

For peer review only

Strength and limitations of this study

- These proposed studies will be unique because we will quantitatively provide an understanding of the social determinants of health (SDoH) that drive health inequalities for children under-five year olds (0-4 years) living in Nairobi and Dhaka slums within intersectionality framework using MAIHDA approach.
- We will use Nairobi Cross-sectional Survey 2012 (NCSS 2012) and Urban Health Survey (UHS 2013) which are one of the few slum surveys in the global south which contain SDoH that shape health inequalities among urban dwellers.
- The passage of time since conduct of NCSS 2012 and UHS 2013: the data are over nine years old and will need to be interpreted cautiously due the dynamic nature of slums.

For peer review only

Introduction

People in urban informal settlements also known as slums face disproportionally worse health conditions compared to other urban dwellers (1). Slum areas are characterised by inadequate access to safe water, inadequate access to sanitation and other infrastructure, poor structural quality of housing, overcrowding, and insecure residential status (2). Health conditions are shaped by Social Determinants of Health (SDoH) - characteristics in which living takes place (3). Critically, SDoH help shape social hierarchies that in turn determine the distribution of power, prestige, and resources among groups in society (4). Slum dwellers are usually excluded and marginalised from formal social systems and structures of power due to legal informality of their dwellings, which denies them rights to access resources, and in turn leads to health inequities (1, 5). Health inequities are differences in health outcomes and in the distribution of health resources experienced between different population groups due to their differences in SDoH (6). Empowerment of slum dwellers coupled with investments in health systems and infrastructure is required to reduce health inequities particularly amongst socially vulnerable groups (7). In turn, this makes empowered slum dwellers accountable because they have increased collective control over the factors that shape their health.

Lack of data that represent the population in slums across cities has been identified as a major hindrance to answering questions critical to the health needs of the slum. In turn, this has created obstacles to understanding the health inequities in slum areas for the effective urban health programming by local governments and other stakeholders (8, 9). Currently, 22.8% of the world's population live in slums, and over 90% of slum dwellers live in low and middle income Countries (LMICs), including hundreds of millions of children (10, 11). Aggregated statistics show that child mortality and health outcomes in rural and urban areas in low and middle income countries have improved between 2000 and 2014 (12). However, during the same period, studies have shown that children in slums tend to experience worse health outcomes than other urban and rural areas (8, 12-16). This is because slums are known to have poor services including water drainage, lack of piped water, flooding, poor sewerage, and housing challenges such as overcrowding which are risk factors for waterborne and vector-borne diseases (17-19). For example, infants who live in slums without piped water may have been shown to experience up to 4.8 fold higher rates of death from diarrhea (18). Since the health of children, particularly those under five years old (i.e. 0-4 years), depends greatly on health and wellbeing of their mothers and families and the broad SDoH factors of where they live; it is crucial to understand how these factors intersect to create complex and unique positions of vulnerability for children in slums(17). To fill the knowledge gap and inform such action, we will systematically examine how various SDoH factors intersect with individual factors to affect the health of under-five children living in informal settlements in (LMICs), through an

For peer review only - http://bmjopen.bmj.com/site/about/guidelines.xhtml

intersectionality lens. The health conditions which will be considered include diarrhea, cough, fever, and acute respiratory infection (ARI) which mostly affect under-five children in slums(20).

SDoH are defined based on the commission for social determinants of health (CDSH) framework which classifies them into structural and intermediary determinants (3, 21). Structural determinants refer to those factors that generate or reinforce stratification in society by exerting a powerful influence on power, prestige, and access to resources and thereby influencing people's health (3). Structural factors include income, age, education, occupation, gender, race/ethnicity, sexuality, disability, and social class. On the other hand, intermediary SDoH are those factors which structural determinants operates through to shape health outcomes of individuals and are grouped into four main categories. They are: 1) material circumstances (e.g., housing and neighborhood quality, consumption potential, and physical work environment), 2) psychosocial circumstances (i.e., relationships, social support, and coping styles), 3) behavioral/biological circumstances (i.e., nutrition, physical activity, tobacco consumption, alcohol consumption, substance abuse), and 4) health system, particularly access to health care. Any attempts to address health inequalities especially among the vulnerable and marginalised must focus on understanding how these multiple SDoH interact with individual factors to shape health inequalities.

An intersectionality lens provides a systematic approach to examining every person's health outcome as fundamentally different from those of others, based on their unique positioning within a web of interacting social determinants (3, 22-25). It assumes that various SDoH interact and change through time to present unique circumstances for individuals or population groups. Therefore intersectionality allows us to account for the complexity of the real world in understanding how different SDoH influence health inequities through marginalisation and privilege in multiplicative and interactional ways based on the lived realities of different groups, without the need to make prior assumptions regarding the importance of one or multiple social categories (24, 26). This is achieved by using Multilevel Analysis of Individual Heterogeneity and Discriminatory Accuracy (MAIHDA) that aims to identify intersectional effects by identifying social groups from multiple interactions that are discriminated and disadvantaged than would be expected in absence of these interactions (27-30). Moreover, an intersectional approach enables will us to understand the drivers of health inequities in the context of how individual identities interact with SDoH to promote/rectify health inequalities in dynamic ways in among under-five living in slums (23, 24).

For peer review only - http://bmjopen.bmj.com/site/about/guidelines.xhtml

For peer review only

Aim

We explore how SDoH influence under-five children health conditions among dwellers in slums within an intersectionality framework. The findings will inform how individual and social inequities are shaped, and what action can be taken to offset burdens in terms of effective policy and programme development for vulnerable under-five children.

Objective

The primary analytic objective is to systematically examine how various SDoH, and individual factors affect health conditions (i.e., diarrhea, fever, cough, and ARI) of under-five children in slums within an intersectionality framework.

Data

In the proposed study, separate analyses and papers applying the same statistical methods for Bangladesh and Kenya are planned. This is informed by the differences in health priorities and contexts across cities (i.e., Dakar and Nairobi) resulting in two distinct publications for Bangladesh and Kenya. These studies will use cross-sectional data from the Nairobi Cross-section Slums Survey (NCSS 2012) for Kenya and Urban Health Survey 2013 (UHS 2013) for Bangladesh, as these are the current disaggregated datasets for slum surveys in both Bangladesh and Kenya. Disaggregated datasets for secondary data analyses are not available in other countries (India and Sierra Leone), where field activities for ARISE project are also being implemented.

The choice of the SDoH characteristics to be included as explanatory variables in the planned analyses will be informed by the literature on the factors that influence health conditions for under-five and discussions with researchers from where the data were collected (12, 20, 31-36). These variables include age and sex for under-fives which have been shown to be determinants of childhood morbidity (12, 32). Moreover, under-five health conditions are closely related to the structural factors such as age and education of head of households and mothers since they affect their ability to provide safe places to grow and live and the ability of households to adopt preventive strategies at a given time (20, 33, 35). The poor hygiene practices in slums which are associated with poor water drainage, inadequate access to safe water, open sewers, and overcrowding also exacerbates health conditions for under-five (37). Finally, higher levels of malnutrition and lower immunisation coverage among under-five living in slums leads to their poor health (31, 38-41).

Nairobi Cross-section Slums Survey (NCSS 2012)

The NCSS 2012 data were collected by the African Population and Health Research Center (APHRC) from all informal settlements in Nairobi between June and November 2012 (8). The sample to be included in the survey was calculated based on the percentage of under-five children with diarrhea

For peer review only

in the two weeks preceding to the survey using a margin of error of 0.03, design effect of 1.50 and critical value of α=0.05 (8). A two-stage random sampling methodology was used and a total of 5,490 households and 4,420 women aged 12-49 years were successfully interviewed yielding a response rate of 88 and 86 percent respectively. We are interested in women because their questionnaire contained a module on child's health, where we are to obtain our health outcomes of interest. Participation was voluntary and no compensation or financial incentive was offered. A total of 2,199 children's data aged under 5 years were provided in the women's questionnaire.

In this study we consider three health conditions for children: 1) whether a child had fever or not, 2) whether a child had cough or not, and 3) whether a child had diarrhea or not. The explanatory variables to be considered in the analysis are classified into four categories: 1) children's demographics (i.e., age and sex), head of household characteristics (i.e., sex, ethnic group, education and age), 3) child's mother characteristics (i.e. age) and 4) social structure characteristics in the household (i.e., wealth index, length of stay, religion, education, tenure, food availability, health insurance, income generating activity, disability and catastrophic health costs) (see Figure 1). Catastrophic health expenditure will be computed using the empirical methodological procedure used by (42) and we will take a 40% threshold which is informed by (43). The wealth index was generated by data provider using source of drinking water, type of toilet facility, cooking fuel used, lighting type at night, material used to construct floor, wall and roof of dwelling, and household possessions (ownership of household items) (8) (see Figure 1).

Figure 1

Bangladesh Urban Health Survey (UHS 2013)
The Urban Health Survey (UHS) 2013 is a representative cross-sectional household survey implemented jointly by National Institute of Population Research and Training (NIPORT), Measure Evaluation, University of North Carolina at Chapel Hill, USA, icddr,b and Associates for Community and Population Research, a Bangladeshi private research agency (44). The survey collected information designed to examine intra-urban differentials in heath and service utilisation from 53,790 households. These households were selected using a stratified three-stage sampling procedure in three urban domains: 1) City corporation slum, 2) City corporation non-slum, and 3) other urban areas. The sample was calculated based on the percentage of all births in the three years preceding the date when the survey was delivered using a margin of error of 0.03 which is equivalent to 24% relative difference (95% CI of 20.6-27.4) (44). Participation was voluntary and no compensation or financial incentive was offered. The proposed analysis will only include the domain

For peer review only - http://bmjopen.bmj.com/site/about/guidelines.xhtml

of city corporation slum since our interest involves investigating social processes which drive health inequalities in informal settlements. In addition, we will consider the women subsample from the survey because their questionnaire contained a module on child's health and nutrition. The number of households selected in the domain of city corporation slum were 15,750 and those interviewed 14,806 yielding a response rate of 94 percent. A total of 14,702 women were eligible for interview and 14,011 were interviewed yielding a 95% response rate.

We will consider three child health conditions: 1) whether a child had fever or not, 2) whether a child had cough or not, and 3) whether a child had an acute respiratory infection (ARI) or not. ARI is a cough accompanied by short, rapid, or difficult breathing which is chest related and usually considered as a proxy for pneumonia. Diarrhea will not be considered because it is not available in the UHS 2013. The explanatory variables to be considered in the analyses will be classified into four categories: 1) children's demographics (i.e., age and sex), 2) mother's demographics (i.e., religion, age, highest level of education, employment status, ever attended school, and marital status), 3) head of household demographics (i.e., sex, and age) and 4) social structure characteristics in the household (i.e., wealth index, dwelling ownership, land ownership, cooking fuel, garbage disposal method, kitchen type, house type and division) (see Figure 2). Wealth index was constructed by data provider using principal components analysis (PCA) based on the following variables: dwelling characteristics such as presence of electricity, type of water source, type of toilet, and floor, wall, and roof material, household ownership of selected assets and durable goods (radio, television, motorcycle, computer, refrigerator, electric fan, and automobile), and two indicators of housing tenure (whether the household held title to the dwelling and/or the land).

Figure 2

Table 1 presents a summary of variables which will be considered for analyses for both NCSS 2012 and UHS 2013. The differences in outcome and explanatory variables in NCSS 2012 and UHS 2013 also informed the need for separate analyses for Kenya and Bangladesh.

For peer review only - http://bmjopen.bmj.com/site/about/guidelines.xhtml

Table 1: List of outcome and explanatory variables for both NCSS 2012 and UHS 2013

	NCSS 2012	UHS 2013
	Variables	Variables
Health conditions	diarrhea, fever, cough	fever, cough, acute respiratory infection (ARI)
Under-five demographics	age, sex	age, sex
Head of household characteristics	age, sex, education, ethnic group	age, sex, education, marital status
Child's mother characteristics	age	age, marital status, ever attended school, highest education, employment, religion
Social structural characteristics	wealth index, length of stay, religion, income generating activity, tenure, disability, food availability, health insurance and health catastrophic costs	wealth index, dwelling ownership, land ownership, garbage disposal, cooking fuel, kitchen type, migration status, house type and division

Statistical methods

The effects of SDoH on children's health outcomes in informal settlements through an intersectionality lens will be assessed using inter and intra categorical analysis technique known as multilevel analysis of individual heterogeneity and discriminatory accuracy (MAIHDA) (19-21). MAIHDA aims to primarily identify intersectional effects by identifying groups that are advantaged than would be expected in the absence of interaction by distinguishing between additive and intersectional/multiplicative effects (28, 45). This allows consideration of both group averages and differences/variation within and between these groups by decomposing the mean outcome for any intersectional group into the additive effects of attribute at each intersection, and additional intersectional effect specific to that group.

MAIHDA therefore, allows multiplicative modelling of health inequalities at the intersection of multiple SDoH by analysing the heterogeneity (i.e. differences) within and between intersectional groups/strata by separating variance (i.e. the measure of variation) into – the between-strata (i.e. differences across strata) and the within - strata (i.e. differences of individuals within a given stratum) (45). MAIHDA also allows quantification of discriminatory accuracy (DA) of the intersectional group by discerning those who have an outcome of interest from those who don't

For peer review only - http://bmjopen.bmj.com/site/about/guidelines.xhtml

For peer review only

using Variance Partitioning Coefficient (VPC) and the area under the receiver operating characteristic curve (AUC-ROC) (27, 28, 45). VPC will quantify the share of the total individual variance in having a health condition that is accounted for at the intersectional strata level with values higher than 5% indicating an acceptable DA (46, 47). On the other hand, AUC-ROC measures the ability of the model to classify individuals with or without health condition as a function of individual's predicted probabilities and is and is bounded between 0.5 and 1 (46, 47) (see (Supplemental file) for details). A value of 0.5 indicates that model predictions are no better than random guessing meaning that predictor variables used in the model have no predictive power, while a value of 1 represents perfect discrimination between under-five with or without health condition (49). In our proposed analyses, AUC-ROC values greater than 0.7 and VPC greater than 5% will indicate an acceptable DA and existence of intersectional effects.

For peer review only

The advantage of MAIHDA is that we will look at intersectionality as a mix of both marginalisation and privilege (27, 29, 45). Generally, an interaction-based fixed effects approach looks at intersectionality from the perspective of marginalisation only, which runs the risk of reinforcing the notion of social dominance of the privileged groups which are used as "default" categories. In addition, from an analytical perspective, multilevel models do not face the issues of scalability (i.e., a model's inability to accommodate an increase in the number of variables included), model parsimony (i.e., a simple model not having great explanatory predictive power), and reduced sample size in some intersectional groups (which influences whether an effect size is determined or not) (28, 48). If desired, we can extend this multilevel framework into a multivariate multilevel model to analyse more than two health outcomes simultaneously, especially for health conditions which occur concurrently affecting other aspects of life (49, 50).

Multilevel models which are mainly used when modelling clustering of individuals based on some shared attributes such as neighborhood, school, or household, among others. The adoption of multilevel models is therefore motivated by the fact that clustering of individuals can also include abstract groupings such as a set of SDoH associated with their intersectional social identities and individual characteristics. As an illustrative example, consider investigating the effects of child's age (i.e., up to 1 year, 2 to 3 years., 4 to 5 years), child's sex (i.e., female or male), head of household education (none, primary, secondary and higher), sex of the head of household (i.e., male or female), age of the head of household (i.e., 18-25 years, 26-32 years, 33-40 years, >40 years) and household's health insurance status (yes or no). Using the six variables and their corresponding categories: child's age (3), child's sex (2), head of household education (3), head of household sex (2), head of household age (4) and household's health insurance status (2) we can create 288 intersectional groups/strata. The first stratum can consist of a child who is a female, aged up to 1

For peer review only - http://bmjopen.bmj.com/site/about/guidelines.xhtml

year and less, coming from a household whose head is a female, aged 18-25 years and with no education and the household is not covered by health insurance, and the process continues until all the children at level 1 are nested within the groups/strata ($N = 288$) at level 2. This implies that children at level 1 who share similar SDoH factors end up being in the same intersectional group/stratum at level 2.

To capture differences in health disparities in children health conditions between different groups, for example - males and females, we can assess child's sex-specific effects through their interactions with child 's age, head of household's sex, age and education and household's health insurance using interaction-based fixed model. However, interaction -based fixed effects model formulation only addresses interactions between child's sex and each of the other five variables and not all interactions (Supplemental file) Eq. 3. If we were to consider all possible interactions among the six variables, we will have 288 interaction terms in the regression model which may result in the model having parsimony and scalability issues due to geometrical growth of coefficients as more variables are included in the model. In addition, it would be difficult to interpret the results and reduced sample size in some interaction groups may influence whether an association is determined or not (28, 48, 50). We will overcome this limitation of interaction-based fixed effects models by applying MAIHDA approach (28). This involves treating social strata/groups defined by child's age and sex, head of household sex, age and education and household's health insurance status as strata which will used to explain whether health inequalities are shaped by different characteristics in each stratum.

Using MAIHDA approach, we will capture the unique interaction/intersectional effect for each social group/stratum (i.e., social strata -specific differences in child's diarrhea) while accounting for sample size differences for each social stratum/group by fitting two successive multilevel logistic regression models (28, 45). The first model 1 will be used to assess whether there is significant clustering within intersectional strata (Supplemental file) Eq. 6. Model 1 will not include any predictor variables and will only have an intercept to estimate the mean health condition and a random effect to model intersectional strata differences (i.e., variance).

Model 2 will be used to explore to which extent intersectional strata differences will be explained by SDoH used in constructing intersectional groups. Model 2 will be an extension of model 1 and will involve adjusting for variables used in constructing intersectional strata as fixed effects (Supplemental file) Eq. 7. Fixed effects in model 2 will be used to estimate model regression coefficients which will be presented as odds ratio and will describe the association between SDoH variables and under-five health conditions. In the absence of intersectional strata differences, fixed

For peer review only - http://bmjopen.bmj.com/site/about/guidelines.xhtml

effects used to construct strata are expected to completely explain intersectional strata differences obtained in model 1 implying that strata random effects in model 2 will be equal to zero.

However, if strata random effects in model 2 are not equal to zero and assuming no relevant variables are omitted on the model it will indicate existence of intersectional effects. This will imply that under-five children living in slums from certain intersectional groups are more vulnerable to adverse health conditions compared to other groups within an already marginalised slum population. To assess the proportion of variance explained by the adding fixed effects in model 2 we will compute the proportional change in variance (PCV) of intersectional strata between models 1 and 2 (Supplemental file) Eq. 9. In the absence of any stratum specific interactions, the fixed effects which will used to construct intersectional strata will completely explain the between stratum variance and all stratum random effects will be equal to zero.

These models 1 and 2 can even be extended to include more than two health conditions in a multivariate multilevel model (49, 50). This model explicitly evaluates the covariance (i.e., a measure of joint variability of two random variables) between different social strata and health conditions which allows us not only to draw conclusions about social group-specific differences but also correlations between health conditions (Supplemental file) Eq. 11. This is desirable since we will assume that they capture related, though distinct, health constructs.

Limitation of this proposed studies is that datasets which will be used were collected over nine years ago (8, 44). Considering the dynamic nature of slums, a more recent data would have been more informative of the SDoH factors which affect under-five health conditions. Despite this, we expect the findings which will obtained to be of great value since these datasets come from the most recently conducted slum surveys in both Bangladesh and Kenya.

Inclusion and exclusion criteria

The analysis will be based on complete case analyses. The choice of variables to be included in constructing intersectional strata for each health condition will be selected by undertaking univariate analyses (51). Only variables that will be significant ($p<0.05$) in the univariate analyses will be used to construct intersectional strata with any correlations assessed using Cramér's V to avoid multicollinearity (52).

Patient and public involvement

There will be no patient or public involvement in this study, as it is based on secondary data.

State date of the analyses

September 2021

Anticipated end date

March 2022

For peer review only - http://bmjopen.bmj.com/site/about/guidelines.xhtml
For peer review only

Ethics and dissemination

The study will use secondary data from the Nairobi Cross-sectional Survey 2012 (NCSS 2012) and Urban Health Survey (UHS 2013) which excludes any participant identifiers. Ethical approval for the NCSS 2012 study was obtained from the Kenya Medical Research Institute's Ethics Review Committee (8) . For UHS 2013, ethical approval was obtained from the Bangladesh Medical Research Council (BMRC) and the Institutional Review Board (IRB) at the School of Public Health, University of North Carolina at Chapel Hill (44). This work as part of ARISE will be used in shaping actions to improve slum health for under five in Bangladesh and Kenya (53). Finding from these studies will be in published peer reviewed journals and presented in international conferences. Analyses will be presented to policy makers and stakeholders of slum health throughout the course of ARISE project.

Data availability

NCSS 2012 data available from African Population Health Centre (APHRC) microdata portal upon reasonable request https://aphrc.org/microdata-portal/ . The UHS 2013 are publicly available at the website of University of North Carolina dataverse portal upon responsible request https://dataverse.unc.edu

Supplementary materials

(Supplemental file)

Ethics statements

Patient consent for publication

Not required

Twitter: @e_kibuchi

Contribution

EK, LG and AL conceived and designed the study. EK wrote the first draft and revised the protocol. PB, IC, NS, PPH, MIHM, CK, ZQ, LW, LD, RF, TS, BA, VS, SG, SS, IG, JKL, BM, HE, AL, and LG critically revised the manuscript.

Funding

The GCRF Accountability for Informal Urban Equity Hub ('ARISE') is a UKRI Collective Fund award with award reference ES/S00811X/1. The MRC/CSO Social and Public Health Sciences Unit is funded by the Medical Research Council (MC_UU_00022/2) and the Scottish Government Chief Scientist Office (SPHSU17).

For peer review only

Disclaimer: The funders have no role in study design or writing of the report. The views expressed in this article are those of the authors.

Competing interest: None declared.

Figure 1: Children health conditions sociodemographic characteristics in the Nairobi Cross-section slums survey (NCSS) 2012.

Figure 2: Children health conditions and sociodemographic characteristics in the Bangladesh Urban Health Survey (UHS) 2013.

For peer review only

References

1. World Health Organization. A billion voices: listening and responding to the health needs of slum dwellers and informal settlers in new urban settings. World Health Organization; 2005.
2. UN-Habitat. State of the World's Cities 2006/7. New York: United Nations. 2006;292.
3. World Health Organization. A conceptual framework for action on the social determinants of health. 2010.
4. Tarlov AR. Social determinants of health: the sociobiological translation. Health and social organization: Routledge; 2002. p. 87-109.
5. Dovey K, King R. Forms of informality: Morphology and visibility of informal settlements. Built Environment. 2011;37(1):11-29.
6. World Health Organization. Health inequities and their causes: World Health Organisation; 2018 [Available from: https://www.who.int/news-room/facts-in-pictures/detail/health-inequities-and-their-causes.
7. World Health Organization. Our cities, our health, our future: acting on social determinants for health equity in urban settings. Report to the WHO Commission on Social Determinants of Health from the Knowledge Network on Urban Settings. Our cities, our health, our future: acting on social determinants for health equity in urban settings Report to the WHO Commission on Social Determinants of Health from the Knowledge Network on Urban Settings. 2008.
8. APHRC. Population and health dynamics in Nairobi's informal settlements: Report of the Nairobi Cross-sectional Slums Survey (NCSS) 2012. Nairobi: APHRC. 2014(April):1-185.
9. Satterthwaite D, editor Health in urban slums depends on better local data. International Conference on Urban Health, Manchester, UK; 2014.
10. UNICEF. Children in an Urban World: the State of the World's Children 2012. Retrieved July. 2012;24:2012.
11. United Nations. World urbanization prospects: The 2018 revision: UN: Department of Economic Social Affairs. Population Division; 2019.
12. Rutstein S, Staveteig S, Winter R, Yourkavitch J, editors. Urban child poverty, health, and survival in low-and middle-income countries. 2017 International Population Conference; 2017: IUSSP.
13. Kyobutungi C, Ziraba AK, Ezeh A, Yé Y. The burden of disease profile of residents of Nairobi's slums: Results from a Demographic Surveillance System. Population health metrics. 2008;6(1):1-8.
14. Barrett JR. A marked disadvantage: rapid urbanization and mortality of young children in Nigeria. National Institute of Environmental Health Sciences; 2010.
15. APHRC. Population and health dynamics in Nairobi's informal settlements: report of the Nairobi Cross-sectional Slums Survey (NCSS) 2000: African Population and Health Research Center; 2002.
16. UNICEF. Understanding urban inequalities in Bangladesh: a prerequisite for achieving Vision 2021. UNICEF Bangladesh, Dhaka. 2010.
17. Unger A. Children's health in slum settings. Arch Dis Child. 2013;98(10):799-805.
18. Victora CG, Smith PG, Vaughan JP, Nobre LC, Lombard C, Teixeira AMB, et al. Water supply, sanitation and housing in relation to the risk of infant mortality from diarrhoea. International journal of epidemiology. 1988;17(3):651-4.
19. World Health Organization. Hidden cities: unmasking and overcoming health inequities in urban settings: World Health Organization. Centre for Health Development; 2010.
20. Unger A. Children's health in slum settings. Archives of disease in childhood. 2013;98(10):799-805.
21. Commission on Social Determinants of Health. Closing the gap in a generation: health equity through action on the social determinants of health: final report of the commission on social determinants of health: World Health Organization; 2008.
22. Green MA, Evans CR, Subramanian SV. Can intersectionality theory enrich population health research? Social Science & Medicine. 2017(178):214-6.

For peer review only

For peer review only - http://bmjopen.bmj.com/site/about/guidelines.xhtml

23. López N, Gadsden VL. Health inequities, social determinants, and intersectionality. 2016.
24. Sen G, Iyer A. Who gains, who loses and how: leveraging gender and class intersections to secure health entitlements. Social science & medicine. 2012;74(11):1802-11.
25. Homan P, Brown TH, King B. Structural intersectionality as a new direction for health disparities research. Journal of health and social behavior. 2021;62(3):350-70.
26. Larson E, George A, Morgan R, Poteat T. 10 Best resources on… intersectionality with an emphasis on low-and middle-income countries. Health policy and planning. 2016;31(8):964-9.
27. Evans CR, Leckie G, Merlo J. Multilevel versus single-level regression for the analysis of multilevel information: The case of quantitative intersectional analysis. Soc Sci Med. 2020;245:112499.
28. Merlo J. Multilevel analysis of individual heterogeneity and discriminatory accuracy (MAIHDA) within an intersectional framework. Soc Sci Med. 2018;203:74-80.
29. Fisk SA, Mulinari S, Wemrell M, Leckie G, Vicente RP, Merlo J. Chronic obstructive pulmonary disease in Sweden: an intersectional multilevel analysis of individual heterogeneity and discriminatory accuracy. SSM-population health. 2018;4:334-46.
30. Merlo J, Yang M, Chaix B, Lynch J, Råstam L. A brief conceptual tutorial on multilevel analysis in social epidemiology: investigating contextual phenomena in different groups of people. Journal of Epidemiology & Community Health. 2005;59(9):729-36.
31. Agarwal S, Bhanot A, Goindi G. Understanding and addressing childhood immunization coverage in urban slums. Indian pediatrics. 2005;42(7):653.
32. Ernst KC, Phillips BS. Slums are not places for children to live: vulnerabilities, health outcomes, and possible interventions. Advances in pediatrics. 2013;60(1):53-87.
33. Vaid A, Mammen A, Primrose B, Kang G. Infant mortality in an urban slum. The Indian Journal of Pediatrics. 2007;74(5):449-53.
34. Crocker-Buque T, Mindra G, Duncan R, Mounier-Jack S. Immunization, urbanization and slums–a systematic review of factors and interventions. BMC public health. 2017;17(1):1-16.
35. Hussain A, Ali SK, Kvåle G. Determinants of mortality among children in the urban slums of Dhaka city, Bangladesh. Tropical Medicine & International Health. 1999;4(11):758-64.
36. Egondi T, Oyolola M, Mutua MK, Elung'ata P. Determinants of immunization inequality among urban poor children: evidence from Nairobi's informal settlements. International journal for equity in health. 2015;14(1):1-5.
37. UN-HABITAT. The challenge of slums: global report on human settlements, 2003: UN-HABITAT -United Nations Human Settlements Programme Staff; 2003.
38. Fotso J-C. Child health inequities in developing countries: differences across urban and rural areas. International journal for equity in health. 2006;5(1):1-10.
39. Fry S, Cousins B, Olivola K. Health of children living in urban slums in Asia and the near east: Review of existing literature and data. Washington, DC: Environmental Health Project, US Agency for International Development. 2002.
40. Sverdlik A. Ill-health and poverty: a literature review on health in informal settlements. Environment and Urbanization. 2011;23(1):123-55.
41. Butala NM, VanRooyen MJ, Patel RB. Improved health outcomes in urban slums through infrastructure upgrading. Social science & medicine. 2010;71(5):935-40.
42. Buigut S, Ettarh R, Amendah DD. Catastrophic health expenditure and its determinants in Kenya slum communities. International journal for equity in health. 2015;14(1):1-12.
43. Xu K, Aguilar A, Carrin G, Evans D, Hanvoravongchai P, Kawabata K, et al. Distribution of health payments and catastrophic expenditures methodology. 2005. World Health Organization, Discussion Paper. 2017(2).
44. NIPORT ICfDDR, Bangladesh ,, Evaluation M. Bangladesh urban health survey 2013. Final report. NIPORT, ICDDRB, and MEASURE Evaluation Dhaka, Bangladesh and Chapel Hill …; 2015.
45. Evans CR, Williams DR, Onnela JP, Subramanian SV. A multilevel approach to modeling health inequalities at the intersection of multiple social identities. Soc Sci Med. 2018;203:64-73.

For peer review only - http://bmjopen.bmj.com/site/about/guidelines.xhtml

46. Wagner P, Merlo J. Measures of discriminatory accuracy in multilevel analysis. Eur J Epidemiol. 2013;28(1):135.
47. Merlo J, Wagner P, Ghith N, Leckie G. An original stepwise multilevel logistic regression analysis of discriminatory accuracy: the case of neighbourhoods and health. PloS one. 2016;11(4):e0153778.
48. Goldstein H. Multilevel statistical models: John Wiley & Sons; 2011.
49. Baldwin SA, Imel ZE, Braithwaite SR, Atkins DC. Analyzing multiple outcomes in clinical research using multivariate multilevel models. Journal of consulting and clinical psychology. 2014;82(5):920.
50. Snijders TA, Bosker RJ. Multilevel analysis: An introduction to basic and advanced multilevel modeling: sage; 2011.
51. Hosmer Jr DW, Lemeshow S, Sturdivant RX. Applied logistic regression: John Wiley & Sons; 2013.
52. Akoglu H. User's guide to correlation coefficients. Turkish journal of emergency medicine. 2018;18(3):91-3.
53. ARISE. Accountability and Responsiveness in Informal Settlements for Equity 2021 [Available from: https://www.ariseconsortium.org/arise-a-new-project-to-improve-accountability-for-health-in-urban-informal-settlements/.

For peer review only

For peer review only - http://bmjopen.bmj.com/site/about/guidelines.xhtml

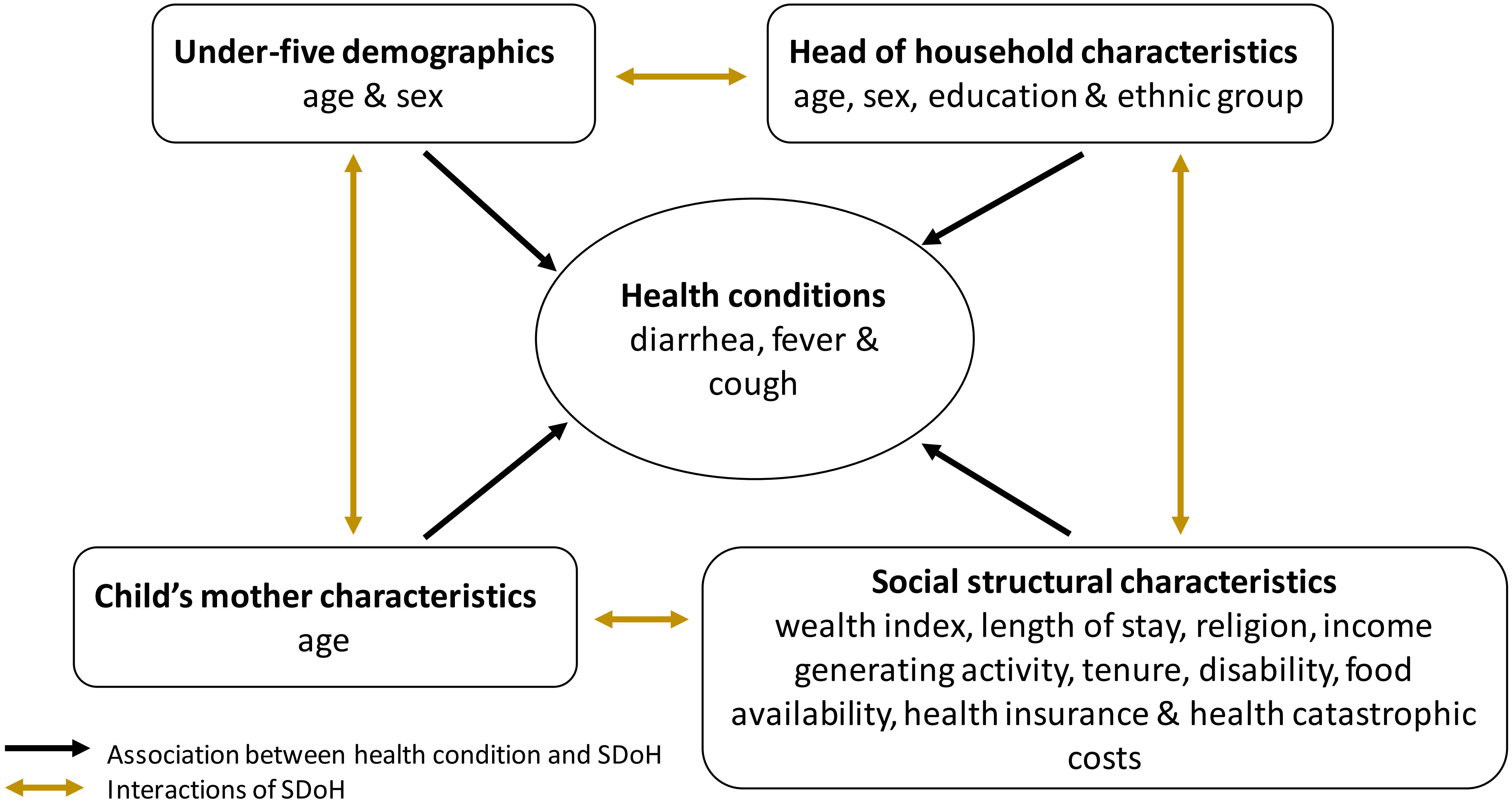
Under-five demographics
age & sex
Head of household characteristics
age, sex, education & ethnic group
Health conditions
diarrhea, fever & cough
Child's mother characteristics
age
Social structural characteristics
wealth index, length of stay, religion, income generating activity, tenure, disability, food availability, health insurance & health catastrophic costs
Association between health condition and SDoH
Interactions of SDoH

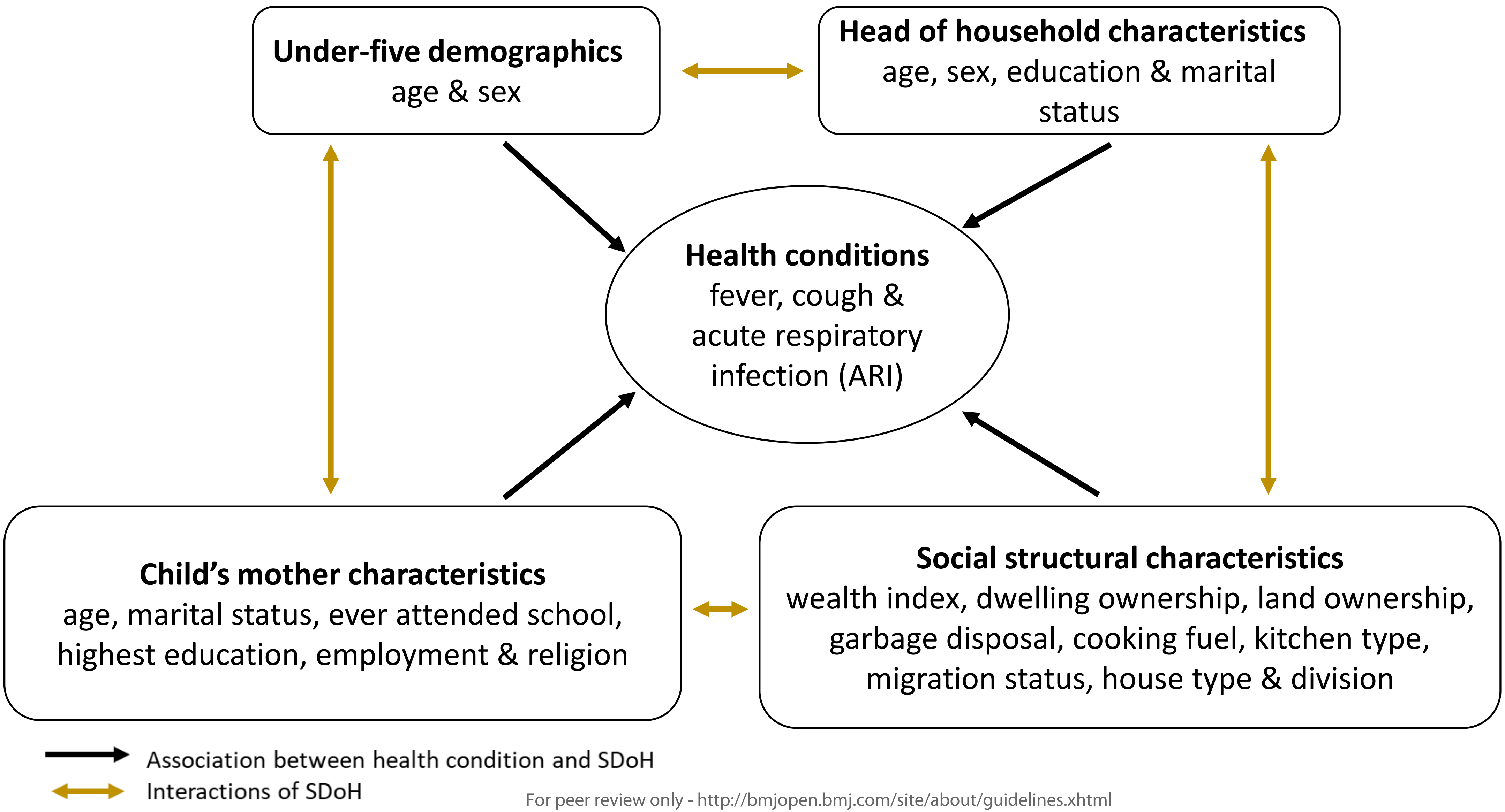
Under-five demographics
age & sex
Head of household characteristics
age, sex, education & marital status
Health conditions
fever, cough & acute respiratory infection (ARI)
Child's mother characteristics
age, marital status, ever attended school, highest education, employment & religion
Social structural characteristics
wealth index, dwelling ownership, land ownership, garbage disposal, cooking fuel, kitchen type, migration status, house type & division
Association between health condition and SDoH
Interactions of SDoH

Supporting information: Statistical methodology details

Let us consider the case where we are interested in investigating effects of child's age (i.e., up to 1 year and less, 2 to 3 years., 4 to5 years), child's sex (i.e., female or male), head of household sex (i.e., male or female), head of household education (none, primary, secondary and higher), head of household age (i.e.,18-25 years, 26-32 years, 33-40 years., >40 years) and household health insurance (yes or no) on children's diarrhea in slums.

Let y_i denote a binary health outcome (i.e., diarrhea) for child $i(i = 1, \dots, i)$ where,

$$y_i = \begin{cases} 0 \;\; absence\ of\ diarhea \\ 1 \;\; Presence\ of\ diarrhea \end{cases} \quad \text{Eq.(1)}$$

y_i is assumed to follow a Bernoulli distribution, with probabilities $\pi_i = Pr(y_i = 0)$ the probability of child i having no diarrhea and $1 - \pi_i = Pr(y_i = 1)$ the probability of child i having diarrhea. Let X_{1i}represent child sex, X_{2i} represent child's age, X_{3i}represent head of household sex, X_{4i} represent head of household education, X_{5i} represent head of household age and X_{6i} represent household health insurance. These six variables represent our explanatory variables. Logistic regression is appropriate for modelling binary (two category) outcomes such as whether a child has diarrhea or not.

The fixed effects logistic regression model for investigating how child sex, child age, head of household sex, head of household education, head of household age and household health insurance are additively associated with child's diarrhea is represented in equation 1 "Eq. (2)".

$$log\left(\frac{\pi_i}{1-\pi_i}\right) = \beta_0 + \beta_1 X_{1i} + \beta_2 X_{21i} + \beta_3 X_{22i} + \beta_4 X_{3i} + \beta_5 X_{41i} + \beta_6 X_{42i} + \beta_7 X_{51i} + \beta_8 X_{52i} + \beta_9 X_{53i} + \beta_{10} X_{6i} \quad \text{Eq. (2)}$$

Eq. (2) estimates the associations of child sex, child age, head of household sex, head of household education, head of household age and household health insurance with child's diarrhea additively (i.e., explanatory effects) and does not accommodate for interactions with each other. In order, to capture specific effects between different groups, for example, child's sex (i.e., males or females), we can assess sex-specific disparities in diarrhea through their interactions with child age, head of household sex, head of household education, head of household age and household health insurance.

Eq. (2) can thus be expanded to include interaction terms, as presented as follows in Eq. (3):

For peer review only - http://bmjopen.bmj.com/site/about/guidelines.xhtml

$$log\left(\frac{\pi_i}{1-\pi_i}\right) = \beta_0 + \beta_1 X_{1i} + \beta_2 X_{21i} + \beta_3 X_{22i} + \beta_4 X_{3i} + \beta_5 X_{41i} + \beta_6 X_{42i} + \beta_7 X_{51i} + \beta_8 X_{52i} + \beta_9 X_{53i} + \beta_{10} X_{6i} + \beta_{11} X_{21i} X_{1i} + \beta_{12} X_{22i} X_{1i} + \beta_{13} X_{3i} X_{1i} + \beta_{14} X_{41i} X_{1i} + \beta_{15} X_{42i} X_{1i} + \beta_{16} X_{51i} X_{1i} + \beta_{17} X_{52i} X_{1i} + \beta_{18} X_{53i} X_{1i} + \beta_{19} X_{6i} X_{1i} \quad \text{Eq. (3)}$$

whereβ_{11} to β_{19} are interaction coefficients between child's sex and other explanatory variables in Eq. (2). Not only does Eq. (3) allow for an analysis that considers the association of child's sex in getting diarrhea but also uncovers how other factors that create and sustain diarrhea may differ based on the sex of child. However, Eq. (3) only addresses interactions between child's sex and the other five variables and if we were to consider all possible interactions among the six variables, we will have a total of 288 fixed effects in the logistic model. The higher number of fixed effects may lead to issues with scalability (i.e., a model's inability to accommodate an increase in the number of variables included), model parsimony (i.e., a simple model not having great explanatory predictive power) and reduced sample size in some intersectional groups which may influence whether an effect is determined is determined or not. In addition, it would be difficult to interpret 288 fixed effects. We can overcome these issues by using multilevel analysis of individual heterogeneity and discriminatory accuracy (MAIHDA) approach.

Multilevel analysis of individual heterogeneity and discriminatory accuracy (MAIHDA)

Now, let us consider the case where we are interested in investigating effects child's age (i.e., up to 1 year and less, 2 to 3 years., 4 to5 years), child's sex (i.e., female or male), head of household sex (i.e., male or female), head of household education (none, primary, secondary and higher), head of household age (i.e.,18-25years, 26-32years, 33-40 years, >40years) and household health insurance (yes or no) on the outcome diarrhea using MAIHDA (1-3). The first step in this approach involves creating groups/strata based on the categories of the social determinants factors we are interested in. This means that children at level 1 who share similar categories of social determinants factors will end up being in the same group/strata at level 2. Therefore, we will have individuals at level 1 nested within 288 groups at level 2.

Therefore, let y_{ij} denote a binary health outcome (i.e., diarrhea) for child $i(i = 1, \dots, n)$ in groups $j\ (j = 1, \dots, N)$ where,

$$y_{ij} = \begin{cases} 0 & absence\ of\ diarrhea \\ 1 & Presence\ of\ diarrhea \end{cases} \quad \text{Eq.(4)}$$

y_{ij} is assumed to follow a Bernoulli distribution, with probabilities $\pi_{ij} = Pr(y_{ij} = 0)$ the probability of child i from group j having no diarrhea and $1 - \pi_{ij} = Pr(y_i = 1)$ the probability of child i from group j having diarrhea. Let X_{1ij} represent child sex, X_{2ij} represent child's age, $X_{3ij}j$represent head

For peer review only - http://bmjopen.bmj.com/site/about/guidelines.xhtml

of household sex, X_{4ij} represent head of household education, X_{5ij} represent head of household age and X_{6ij} represent household health insurance.

The fixed effects logistic regression model for investigating how child sex, child age, head of household sex, head of household education, head of household age and household health insurance are additively associated with child's diarrhea are additively associated with diarrhea can be extended to incorporate groups/strata as represented in Eq. (6)

$$log\left(\frac{\pi_{ij}}{1-\pi_{ij}}\right) = \beta_0 + \beta_1 X_{1ij} + \beta_2 X_{21ij} + \beta_3 X_{22ij} + \beta_4 X_{3ij} + \beta_5 X_{41ij} + \beta_6 X_{42ij} + \beta_7 X_{51ij} + \beta_8 X_{52ij} + \beta_9 X_{53ij} + \beta_{10} X_{6ij} \quad \text{Eq. (5)}$$

Equation 5 can be extended by taking the MAIHDA approach {Merlo, 2018 #375} by treating social groups defined by fixed effects (i.e. child sex, child age, head of household sex, head of household education, head of household age and household health insurance) as intersectional strata which are associated multiplicatively with a child developing diarrhea or not. In this model we exclude all fixed effects. Then we have:

$$log\left(\frac{\pi_{ij}}{1-\pi_{ij}}\right) = \beta_0 + \mu_{0j} \quad \text{Eq. (6)}$$

where β_0 is the intercept and $\mu_{0j} \sim N(0, \sigma^2_{group})$ represents the random intercept for the intersectional stratum level residual which is normally distributed with mean 0 and variance σ^2_{μ}. Eq. (6) include predictor variables, so the intersectional stratum random effect captures both the main effects of SDH used to define intersectional strata and their interactions. Assuming no omitted variable bias, the intersectional strata level residual μ_{0j} captures the unique interaction effect for each intersectional strata (i.e., intersectional -specific differences in health condition) while accounting for sample size differences for each social group.

Equation 6 can be extended into Eq. 7 by including fixed effects (i.e., child sex, child age, head of household sex, head of household education, head of household age and household health insurance) used in construction intersectional strata) as predictor variables and takes the form. Therefore Eq. (7) takes the form

$$log\left(\frac{\pi_{ij}}{1-\pi_{ij}}\right) = \beta_0 + \beta_1 X_{1ij} + \beta_2 X_{21ij} + \beta_3 X_{22ij} + \beta_4 X_{3ij} + \beta_5 X_{41ij} + \beta_6 X_{42ij} + \beta_7 X_{51ij} + \beta_8 X_{52ij} + \beta_9 X_{53ij} + \beta_{10} X_{6ij} + \mu_{0j} \quad \text{Eq. (7)}$$

where β_0 is the intercept and $\mu_{0j} \sim N(0, \sigma^2_{group})$ represents the group level residual which is normally distributed with mean 0 and variance σ^2_{group}. Assuming no omitted variable bias, the group level

For peer review only - http://bmjopen.bmj.com/site/about/guidelines.xhtml

residual μ_{0j} captures the unique interaction effect for each social group/strata (i.e. social groups - specific differences in diarrhea) while accounting for sample size differences for each social group. The relevance of the intersectional strata for understanding individual heterogeneity is evaluated using Variance partitioning Coefficient (VPC) also known as intraclass coefficient (which also informs on the discriminatory accuracy of the intersectional categorisation for distinguishing children with diarrhea from those without (4, 5).

$$VPC = \left(\frac{\sigma^2_{group}}{\sigma^2_{group} + 3.29}\right) \quad \text{Eq. (8)}$$

where the value 3.29 is the variance for the standard logistic distribution (6).

The proportion of variance explained by the adding fixed effects is estimated by calculating the proportional change in variance (PCV) of intersectional strata between null model and model including fixed effects (3)

$$PCV = \left(\frac{\sigma^2_{\mu(1)} - \sigma^2_{\mu(2)}}{\sigma^2_{\mu(1)}}\right) \quad \text{Eq. (9)}$$

where $\sigma^2_{\mu(1)}$ and $\sigma^2_{\mu(2)}$ represents the intersectional strata variances in the null model and the model containing main effects respectively. The PCV represents the proportion of the total between-stratum variance of intersectional strata of the null model that is explained after including main effects. In the absence of any stratum specific interactions, the main effects used to construct the intersectional strata would completely explain the between stratum variance and all stratum random effects would be equal to zero (4, 5).

If desirable, this model can be further extended to include more than two health outcomes in a multivariate multilevel model (7). For example, let's assume that respondents (level 2), social group (level 3) j, with h health outcome variables, $y_1, \dots, y_m$ (i.e., $h = 1, \dots, m$) at level 1 and x_{kij} explanatory variables (i.e., $k = 1, \dots, p$). The outcome variable is denoted as y_{hij} which is the outcome on the h'th health variable for respondent i in social group j. Another useful feature of multivariate multilevel model is that we can account for missing health outcomes not recorded for some respondents by assume that missingness is due to missing at random (MAR) (7). That is, conditionally given the observed data, the missingness indicators are independent of the unobserved data (8).

We can define the complete data vector for the m health outcomes by combining them into a single outcome variable y_{hij} as

$$y_{hij} = \left(y_{1ij} \dots y_{mij}\right) \quad \text{Eq. (8)}$$

In addition, we also create dummy variables $d_1, \dots, d_m$ which are defined for $h = 1, \dots, m$,

$$d_{shij} = \{1(h = s) 0(h \neq s) \quad \text{Eq. (9)}$$

For peer review only - http://bmjopen.bmj.com/site/about/guidelines.xhtml

where dummy variable d_h is 1 or 0, depending on whether the data line refers to outcome variable y_h or to one of the other health outcome variables. With these dummies, the random intercept model Eq. (6) for the m health outcomes can be defined as

$$y_{hij} = \sum_{s=1}^{m} \beta_{0s} d_{shij} + \sum_{k=1}^{p} \sum_{s=1}^{m} \beta_{ks} d_{shij} x'_{kij} + \sum_{s=1}^{m} \mu_{0sj} d_{shij} \quad \text{Eq. (10)}$$

where all variables including the constant are multiplied by the dummy variables d_{shij}. $\beta_{0s}$is the intercept, $x'_{kij}$is a vector of social determinants variables with coefficient vector β while μ_{0j} is a group level residual which follow a multivariate normal distribution $\Sigma = cov(\mu_{0j})$. Therefore, Eq. (10) represent the multivariate data in a multilevel approach with level 1 being health outcomes variables indexed by $h = 1, \ldots, m$, level 2 represents respondents $i = 1, \ldots, n_j$, and level 3 represents social group defined by social determinants variables. This set up models explicitly the covariance between different social groups and health outcomes which allows us not only to draw conclusions about social group-specific differences but also correlations between health outcomes are desirable since we assume that they capture related, though distinct, health constructs.

Summary of steps to be involved in fitting MAIHDA model

1. Selecting the variables to be used in creating intersectional strata.
2. Creating intersectional strata.
3. Fitting a multilevel model 1 which contains only the random intercept for intersectional strata with no fixed effects.
4. Fitting a multilevel model 2 by including variables used in constructing intersectional strata as fixed effects in model 1.
5. Compute measures of discriminatory accuracy (i.e., variance partitioning coefficient (VPC) and area under the receiver operating characteristic curve (AUC-ROC)) both model 1 and 2.
6. Use VPC for models 1 and 2 to compute proportional change in variance (PCV).

For peer review only - http://bmjopen.bmj.com/site/about/guidelines.xhtml

References

1. Evans CR, Leckie G, Merlo J. Multilevel versus single-level regression for the analysis of multilevel information: The case of quantitative intersectional analysis. Soc Sci Med. 2020;245:112499.
2. Merlo J. Multilevel analysis of individual heterogeneity and discriminatory accuracy (MAIHDA) within an intersectional framework. Soc Sci Med. 2018;203:74-80.
3. Merlo J, Yang M, Chaix B, Lynch J, Råstam L. A brief conceptual tutorial on multilevel analysis in social epidemiology: investigating contextual phenomena in different groups of people. Journal of Epidemiology & Community Health. 2005;59(9):729-36.
4. Wagner P, Merlo J. Measures of discriminatory accuracy in multilevel analysis. Eur J Epidemiol. 2013;28(1):135.
5. Fisk SA, Mulinari S, Wemrell M, Leckie G, Vicente RP, Merlo J. Chronic obstructive pulmonary disease in Sweden: an intersectional multilevel analysis of individual heterogeneity and discriminatory accuracy. SSM-population health. 2018;4:334-46.
6. Goldstein H. Multilevel statistical models: John Wiley & Sons; 2011.
7. Snijders TA, Bosker RJ. Multilevel analysis: An introduction to basic and advanced multilevel modeling: sage; 2011.
8. Rubin; DB. Inference and Missing Data. Biometrika. 1976;63(3):581-92.

For peer review only

For peer review only - http://bmjopen.bmj.com/site/about/guidelines.xhtml

BMJ Open

Effects of social determinants on children's health in informal settlements in Bangladesh and Kenya through an intersectionality lens – a study protocol

Journal:	*BMJ Open*
Manuscript ID	bmjopen-2021-056494.R2
Article Type:	Protocol
Date Submitted by the Author:	03-May-2022
Complete List of Authors:	Kibuchi, Eliud; University of Glasgow, MRC/CSO Social and Public Health Sciences Unit<br>Barua, Proloy; BRAC, CGSRHR<br>Chumo, Ivy; African Population and Health Research Center<br>Teixeira de Siqueira Filha, Noemia ; University of York,<br>Phillips-Howard, Penelope; Liverpool School of Tropical Medicine, Department of Clinical Sciences<br>Mithu, Md. Imran Hossain; BRAC, CGSRHR<br>Kabaria, Caroline ; African Population and Health Research Center<br>Quayyum , Zahidul ; BRAC University James P Grant School of Public Health,<br>Whittaker, Lana; Liverpool School of Tropical Medicine, Department of Clinical Sciences<br>Dean, Laura; Liverpool School of Tropical Medicine, Department of International Public Health<br>Forsyth, Ross; University of Glasgow, MRC/CSO Social & Public Health Sciences Unit<br>Selim, Tasmiah; BRAC University James P Grant School of Public Health<br>Aktar, Bachera; BRAC University James P Grant School of Public Health<br>Sai, Varun; The George Institute for Global Health India<br>Garimella, Sureka; The George Institute for Global Health India,<br>Saidu, Samuel; COMAHS<br>Gandi , Ibrahim; Centre Of Dialogue On Human Settlement And Poverty Alleviation (CODOHSAPA)<br>Josyula, Lakshmi; The George Institute for Global Health<br>Mberu, Blessing; African Population and Health Research Center<br>Elsey, Helen ; University of York,<br>Leyland, Alastair; University of Glasgow, MRC/CSO Social and Public Health Sciences Unit<br>Gray, Linsay; University of Glasgow, MRC/CSO Social and Public Health Sciences Unit
**Primary Subject Heading**:	Epidemiology
Secondary Subject Heading:	Global health, Public health
Keywords:	PUBLIC HEALTH, Community child health < PAEDIATRICS, STATISTICS & RESEARCH METHODS, EPIDEMIOLOGY

Effects of social determinants on children's health in informal settlements in Bangladesh and Kenya through an intersectionality lens – a study protocol

Eliud Kibuchi[1], Proloy Barua[2], Ivy Chumo[3], Noemia Siqueira[4], Penelope Phillips-Howard[5], Md Imran Hossain Mithu[2], Caroline Kabaria[3], Zahidul Quayyum[1,2], Lana Whittaker[5], Laura Dean[5], Ross Forsyth[1], *Tasmiah Selim[2], Bachera Aktar[2], Varun Sai[6], Surekha Garimella[6], Samuel Saidu[7], Ibrahim Gandi[8], Joysula k Lakshmi[6], Blessing Mberu[3], Helen Elsey[4], Alastair Leyland[1], Linsay Gray[1]*

[1] University of Glasgow, Glasgow, UK
[2] James P Grant School of Public Health, Brac University, Dhaka, Bangladesh
[3] African Population and Health Research Center, Nairobi, Kenya
[4] University of York, Department of Health Sciences, York, UK
[5] Liverpool School of Tropical Medicine, Pembroke Place, Liverpool, L3 5QA, UK
[6]The George Institute for Global Health, New Delhi, India
[7] COMAHS: University of Sierra Leone, Freetown, Sierra Leone,
[8] Centre Of Dialogue On Human Settlement And Poverty Alleviation (CODOHSAPA), Freetown, Sierra Leone

Corresponding author: Eliud Kibuchi, Eliud.Kibuchi@glasgow.ac.uk, MRC/CSO Social and Public Health Sciences Unit, Institute of Health and Wellbeing, University of Glasgow, Berkeley Square, 99 Berkeley Street, Glasgow, G3 7HR, UK.

Abstract no of words: 286

No of words: 3,515

For peer review only - http://bmjopen.bmj.com/site/about/guidelines.xhtml

Abstract

Introduction

Several studies have shown that residents of urban informal settlements/slums are usually excluded and marginalised from formal social systems and structures of power leading to disproportionally worse health outcomes compared to other urban dwellers. To promote health equity for slum dwellers, requires an understanding of how their lived realities shape inequities especially for young children 0-4 years old (i.e., under-fives) who tend to have a higher mortality compared with non-slum children. In these proposed studies, we aim to examine how key Social Determinants of Health (SDoH) factors at child and household levels combine to affect under-five health conditions, who live in slums in Bangladesh and Kenya through an intersectionality lens.

Methods and analysis

The protocol describes how we will analyse data from the Nairobi Cross-sectional Slum Survey (NCSS 2012) for Kenya and the Urban Health Survey (UHS 2013) for Bangladesh to explore how SDoH influence under-five health outcomes in slums within an intersectionality framework. The NCSS 2012 and UHS 2013 samples will consist of 2,199 and 3,173 under-fives, respectively. We will apply Multilevel Analysis of Individual Heterogeneity and Discriminatory Accuracy (MAIHDA)approach. Some of SDoH characteristics to be considered will include those of children, head of household, mothers, and social structure characteristics of household. The primary outcomes will be whether a child had diarrhea, cough, fever, and acute respiratory infection (ARI) two weeks preceding surveys.

Ethics and dissemination

The results will be disseminated in international peer-reviewed journals and presented in events organized by the Accountability and Responsiveness in Informal Settlements for Equity (ARISE) consortium and international conferences. Ethical approval was not required for these studies. Access to the NCSS 2012 has been given by Africa Population and Health Center and UHS 2013 is freely available

Key words: Informal settlements/slums, under-five, social determinants of health, intersectionality, MAIHDA

For peer review only

For peer review only - http://bmjopen.bmj.com/site/about/guidelines.xhtml

Strength and limitations of this study

- These proposed studies will be unique because we will quantitatively provide an understanding of the social determinants of health (SDoH) that drive health inequalities for children under-five year olds (0-4 years) living in Nairobi and Dhaka slums within intersectionality framework using MAIHDA approach.
- We will use Nairobi Cross-sectional Survey 2012 (NCSS 2012) and Urban Health Survey (UHS 2013) which are one of the few slum surveys in the global south which contain SDoH that shape health inequalities among urban dwellers.
- The passage of time since conduct of NCSS 2012 and UHS 2013: the data are over nine years old and will need to be interpreted cautiously due the dynamic nature of slums.

For peer review only

For peer review only - http://bmjopen.bmj.com/site/about/guidelines.xhtml

Introduction

People in urban informal settlements also known as slums face disproportionally worse health outcomes compared to other urban dwellers (1). Slum areas are characterised by inadequate access to safe water, inadequate access to sanitation and other infrastructure, poor structural quality of housing, overcrowding, and insecure residential status (2). Health outcomes are shaped by Social Determinants of Health (SDoH) - characteristics in which living takes place (3). Critically, SDoH help shape social hierarchies that in turn determine the distribution of power, prestige, and resources among groups in society (4). Slum dwellers are usually excluded and marginalised from formal social systems and structures of power due to legal informality of their dwellings, which denies them rights to access resources, and in turn leads to health inequities (1, 5). Health inequities are differences in health outcomes and in the distribution of health resources experienced between different population groups due to their differences in SDoH (6). Empowerment of slum dwellers coupled with investments in health systems and infrastructure is required to reduce health inequities particularly amongst socially vulnerable groups (7). In turn, this makes empowered slum dwellers accountable because they have increased collective control over the factors that shape their health.

Lack of data that represent the population in slums across cities has been identified as a major hindrance to answering questions critical to the health needs of the slum. In turn, this has created obstacles to understanding the health inequities in slum areas for the effective urban health programming by local governments and other stakeholders (8, 9). Currently, 22.8% of the world's population live in slums, and over 90% of slum dwellers live in low and middle income Countries (LMICs), including hundreds of millions of children (10, 11). Aggregated statistics show that child mortality and health outcomes in rural and urban areas in low and middle income countries have improved between 2000 and 2014 (12). However, during the same period, studies have shown that children in slums tend to experience worse health outcomes than other urban and rural areas (8, 12-16). This is because slums are known to have poor services including water drainage, lack of piped water, flooding, poor sewerage, and housing challenges such as overcrowding which are risk factors for waterborne and vector-borne diseases (17-19). For example, infants who live in slums without piped water may have been shown to experience up to 4.8 fold higher rates of death from diarrhea (18). Since the health of children, particularly those under five years old (i.e. 0-4 years), depends greatly on health and wellbeing of their mothers and families and the broad SDoH factors of where they live; it is crucial to understand how these factors intersect to create complex and unique positions of vulnerability for children in slums (17). To fill the knowledge gap and inform such action, we will systematically examine how various SDoH factors intersect with individual factors to affect the health of under-five children living in slums (LMICs), through an intersectionality lens. The health

For peer review only - http://bmjopen.bmj.com/site/about/guidelines.xhtml

outcomes which will be considered include diarrhea, cough, fever, and acute respiratory infection (ARI) which mostly affect under-five children in slums(20). For example, poor sanitation and lack of safe drinking water makes diarrhea a leading cause of death among children aged below five years, while fevers, coughs, and acute respiratory infections (ARI) are mainly caused by the poor state of housing and overcrowding (16, 20).

SDoH are defined based on the commission for social determinants of health (CDSH) framework which classifies them into structural and intermediary determinants (3, 21). Structural determinants refer to those factors that generate or reinforce stratification in society by exerting a powerful influence on power, prestige, and access to resources and thereby influencing people's health (3). Structural factors include income, age, education, occupation, gender, race/ethnicity, sexuality, disability, and social class. On the other hand, intermediary SDoH are those factors which structural determinants operates through to shape health outcomes of individuals and are grouped into four main categories. They are: 1) material circumstances (e.g., housing and neighborhood quality, consumption potential, and physical work environment), 2) psychosocial circumstances (i.e., relationships, social support, and coping styles), 3) behavioral/biological circumstances (i.e., nutrition, physical activity, tobacco consumption, alcohol consumption, substance abuse), and 4) health system, particularly access to health care. Any attempts to address health inequalities especially among the vulnerable and marginalised must focus on understanding how these multiple SDoH interact with individual factors to shape health inequalities.

An intersectionality lens provides a systematic approach to examining every person's health outcome as fundamentally different from those of others, based on their unique positioning within a web of interacting social determinants (3, 22-25). It assumes that various SDoH interact and change through time to present unique circumstances for individuals or population groups. Therefore intersectionality allows us to account for the complexity of the real world in understanding how different SDoH influence health inequities through marginalisation and privilege in multiplicative and interactional ways based on the lived realities of different groups, without the need to make prior assumptions regarding the importance of one or multiple social categories (24, 26). Quantitatively this can be explored using Multilevel Analysis of Individual Heterogeneity and Discriminatory Accuracy (MAIHDA) approach (27-30). This approach will enable us to understand the drivers of health inequities in the context of how individual identities interact with SDoH to promote/rectify health inequalities in dynamic ways in among under-five living in slums (23, 24).

For peer review only - http://bmjopen.bmj.com/site/about/guidelines.xhtml

For peer review only

Aim

We explore how SDoH influence under-five children health outcomes among dwellers in slums within an intersectionality framework. The findings will inform how individual and social inequities are shaped, and what action can be taken to offset burdens in terms of effective policy and programme development for vulnerable under-five children.

Objective

The primary analytic objective is to systematically examine how various SDoH, and individual factors affect health outcomes (i.e., diarrhea, fever, cough, and ARI) of under-five children in slums within an intersectionality framework.

Data

In the proposed study, separate analyses and papers applying the same statistical methods for Bangladesh and Kenya are planned. The underlying social and living conditions in Dhaka and Nairobi slums are different which necessitates two distinct publications for Bangladesh and Kenya for effective interventions (8, 31-33). These studies will use cross-sectional data from the Nairobi Cross-section Slums Survey (NCSS 2012) for Kenya and Urban Health Survey 2013 (UHS 2013) for Bangladesh, as these are the current disaggregated datasets for slum surveys in both Bangladesh and Kenya. Disaggregated datasets for secondary data analyses are not available in other countries (India and Sierra Leone), where field activities for ARISE project are also being implemented.

The choice of the SDoH characteristics to be included as explanatory variables in the planned analyses will be informed by the literature on the factors that influence health outcomes for under-five (12, 20, 34-39). These variables include age and sex for under-fives which have been shown to be determinants of childhood morbidity (12, 35). Moreover, under-five health outcomes are closely related to the structural factors such as age and education of head of households and mothers since they affect their ability to provide safe places to grow and live and the ability of households to adopt preventive strategies at a given time (20, 36, 38). The poor hygiene practices in slums which are associated with poor water drainage, inadequate access to safe water, open sewers, and overcrowding also exacerbates health outcomes for under-five (40). Finally, higher levels of malnutrition and lower immunisation coverage among under-five living in slums leads to their poor health (34, 41-44).

Nairobi Cross-section Slums Survey (NCSS 2012)

The NCSS 2012 data were collected by the African Population and Health Research Center (APHRC) from all slums in Nairobi between June and November 2012 (8). The sample to be included in the survey was calculated based on the percentage of children 12-23 months who had been fully

For peer review only - http://bmjopen.bmj.com/site/about/guidelines.xhtml

immunized using a margin of error of 0.03, design effect of 1.50 and critical value of α=0.05 (8). The number of households required to estimate the percentage of children 12-23 months fully vaccinated was large enough to allow estimation of the other indicators such as diarrhea, fever, and cough with the specified precision. A two-stage random sampling methodology was used and a total of 5,490 households and 4,420 women aged 12-49 years were successfully interviewed yielding a response rate of 88 and 86 percent, respectively. We will be interested in women because their questionnaire contained a module on child's health, where we are to obtain our health outcomes of interest. Participation was voluntary and no compensation or financial incentive was offered. The 4,420 women participants provided data on 2,199 children aged 5 and under.

In this study we consider three health outcomes for children: 1) whether a child had fever or not, 2) whether a child had cough or not, and 3) whether a child had diarrhea or not. The predictor variables to be considered in the analysis are classified into four categories: 1) children's demographics (i.e., age and sex), head of household characteristics (i.e., sex, ethnic group, education and age), 3) child's mother characteristics (i.e. age) and 4) social structure characteristics in the household (i.e., wealth index, length of stay, religion, education, tenure, food availability, health insurance, income generating activity, disability and catastrophic health costs). Catastrophic health expenditure will be computed using the empirical methodological procedure used by (45) and we will take a 40% threshold which is informed by (46). The wealth index was generated using source of drinking water, type of toilet facility, cooking fuel used, lighting type at night, material used to construct floor, wall and roof of dwelling, and household possessions (ownership of household items) (8). Detailed description of health outcomes and predictor variables for NCSS 2012 are found in (Supplementary file) Table s1.

Bangladesh Urban Health Survey (UHS 2013)

The UHS 2013 is a representative cross-sectional household survey implemented jointly by 1) National Institute of Population Research and Training (NIPORT), 2) Measure Evaluation, University of North Carolina at Chapel Hill, USA, 3) International Centre for diarrhea disease research, Bangladesh (icddr,b)and 4) Associates for Community and Population Research (33). The survey collected information designed to examine intra-urban differentials in heath and service utilisation from 53,790 households. These households were selected using a stratified three-stage sampling procedure in three urban domains: 1) City corporation slum, 2) City corporation non-slum, and 3) other urban areas. The key indicators used to calculate the sample size were (i) under-five mortality and (ii) percentage of birth deliveries in the health facilities for all births in the last three years (33). Participation was voluntary and no compensation or financial incentive was offered. The proposed

For peer review only - http://bmjopen.bmj.com/site/about/guidelines.xhtml

analysis will only include the domain of city corporation slum since our interest involves investigating social processes which drive health inequalities in slums. In addition, we will consider the women subsample from the survey because their questionnaire contained a module on child's health and nutrition. The number of households selected in the domain of city corporation slum were 15,750 and those interviewed 14,806 yielding a response rate of 94 percent. A total of 14,702 women were eligible for interview and 14,011 were interviewed yielding a 95% response rate.

We will consider three child health conditions: 1) whether a child had fever or not, 2) whether a child had cough or not, and 3) whether a child had an acute respiratory infection (ARI) or not. ARI is a cough accompanied by short, rapid, or difficult breathing which is chest related and usually considered as a proxy for pneumonia. The predictor variables to be considered in the analyses will be classified into four categories: 1) children's demographics (i.e., age and sex), 2) mother's demographics (i.e., religion, age, highest level of education, employment status, ever attended school, and marital status), 3) head of household demographics (i.e., sex, and age) and 4) social structure characteristics in the household (i.e., wealth index, dwelling ownership, land ownership, cooking fuel, garbage disposal method, kitchen type, house type and division) (see Figure 1). Wealth index was constructed by data provider using principal components analysis (PCA) based on the following variables: dwelling characteristics such as presence of electricity, type of water source, type of toilet, and floor, wall, and roof material, household ownership of selected assets and durable goods (radio, television, motorcycle, computer, refrigerator, electric fan, and automobile), and two indicators of housing tenure (whether the household held title to the dwelling and/or the land). A detailed description of variables and their categorical levels for UHS 2013 are presented in (Supplementary file) Table s2.

Table 1 presents a summary of variables which will be considered for analyses for NCSS 2012 and UHS 2013.The differences in outcome and predictor variables in NCSS 2012 and UHS 2013 also informed the need for separate analyses for Kenya and Bangladesh. Data on ARI and diarrhea were not available in the NCSS 2012 and UHS 2013, respectively. A causal diagram showing the direct pathway between the four categories of variables and under-five health outcomes is shown in Figure 1.

Figure 1

For peer review only

Table 1: List of outcome and explanatory variables for both Nairobi Cross sectional survey (NCSS) 2012 and Bangladesh Urban Health Survey (UHS)2013

	NCSS 2012	UHS 2013
	Variables	Variables
**Outcomes**		
Health outcomes	diarrhea, fever, cough	fever, cough, acute respiratory infection (ARI)
**Predictors**		
Under-five demographics	age, sex	age, sex
Head of household characteristics	age, sex, education, ethnic group	age, sex, education, marital status
Child's mother characteristics	age	age, marital status, ever attended school, highest education, employment
Social structural characteristics	wealth index, length of stay, religion, income generating activity, tenure, disability, food availability, health insurance and health catastrophic costs	wealth index, dwelling ownership, land ownership, garbage disposal, cooking fuel, having kitchen, migration status, housing type and division

Statistical methods

The effects of SDoH on children's health outcomes in slums through an intersectionality lens will be assessed using multilevel analysis of individual heterogeneity and discriminatory accuracy (MAIHDA) approach developed to analyse intersectional inequalities (27-29, 47). MAIHDA aims to primarily identify intersecting inequalities in a quantitative way by defining intersectional groups according to combinations of social attributes which is like clustering of individuals based on some shared attributes such as neighborhood, school, or household, among others (28, 48). That is, individuals can be clustered based on abstract groupings such as a set of SDoH associated with their intersectional social identities and individual characteristics.

MAIHDA therefore, allows multiplicative modelling of health inequalities at the intersection of multiple SDoH by analysing the heterogeneity (i.e., differences) within and between intersectional groups/strata by separating variance (i.e. the measure of variation) into – the between-strata (i.e. differences across strata) and the within - strata (i.e. differences of individuals within a given stratum) (28, 29).

For peer review only - http://bmjopen.bmj.com/site/about/guidelines.xhtml
For peer review only

The advantage of MAIHDA is that we will look at intersectionality as a mix of both marginalisation and privilege (27, 28) . Generally, an interaction-based fixed effects approach looks at intersectionality from the perspective of marginalisation only, which runs the risk of reinforcing the notion of social dominance of the privileged groups which are used as "default" categories. In addition, from an analytical perspective, MAIHDA models do not face the issues of scalability (i.e., a model's inability to accommodate an increase in the number of variables included), model parsimony (i.e., a simple model not having great explanatory predictive power), and reduced sample size in some intersectional groups (which influences whether an effect size is determined or not) (28). If desired, we can extend this multilevel framework applied in MAIHDA into a multivariate multilevel model to analyse more than two health outcomes simultaneously, especially for health outcomes which occur concurrently affecting other aspects of life (49, 50). MAIHDA estimates intersectional effect in two steps (28, 48).

First, a null model (i.e., model 1) will be specified with individuals at level one nested within social groups at level two to assess whether there is significant clustering within intersectional strata. Model 1 will not include any predictor variables and will only have an intercept to estimate the mean health outcome and a random effect to model intersectional strata differences (i.e., variance). This will allow estimation of the extent to which the variance in an outcome is explained by differences across intersections versus differences within using variance partitioning coefficient (VPC) also known as intraclass coefficient (ICC) and the area under the receiver operating characteristic curve (AUC-ROC) (27, 28, 48). VPC and AUC-ROC measures will also be used to quantify discriminatory accuracy (DA) which is ability of the model to discriminate individuals with and without an outcome of interest (27, 28, 30, 51). VPC quantifies the share of the total individual variance in having an outcome that is accounted for at the intersectional strata level with values higher than 5% indicating an acceptable DA (28, 47, 52). That is, a high VPC indicates that intersections have a substantially different mean levels of an outcome and that individuals within these group are similar, while a low VPC indicates that individuals within an intersectional group differ substantially (47, 51). On the other hand, AUC-ROC measures the ability of the model to classify individuals with or without an outcome as a function of individual's predicted probabilities and is and is bounded between 0.5 and 1 (52, 53) (see for (Supplementary file) details).

In the second step, we will extend model 1 and by adjusting for variables used in constructing intersectional strata as fixed effects (i.e., model 2). Model 2 will be used to explore to which extent intersectional strata differences will be explained by SDoH used in constructing intersectional groups. Fixed effects in model 2 will be used to estimate model regression coefficients which will be presented as odds ratio and will describe the association between SDoH variables and under-five

For peer review only - http://bmjopen.bmj.com/site/about/guidelines.xhtml

health conditions. In the absence of intersectional strata differences, fixed effects used to construct strata are expected to completely explain intersectional strata differences obtained in model 1. That is, VPC values will be around zero in model 2 indicating that intersectional effects are fully explained by fixed effects and are therefore additive and not multiplicative (29, 47). This will indicate absence of any stratum/group specific interactions since the fixed effects used to construct intersectional strata will completely explain the between stratum variance and all stratum random effects.

However, if strata random effects in model 2 are not equal to zero and assuming no relevant variables are omitted on the model it will indicate existence of multiplicative intersectional effects. This will imply that certain intersectional groups are more vulnerable to an outcome of interest health outcomes compared to other groups. To assess the proportion of variance explained by the adding fixed effects in model 2 we will compute the proportional change in variance (PCV) of intersectional strata between models 1 and 2 (see more details (Supplementary file)). The lower the PCV, the higher the amount of unexplained variance which can be due to either interaction effects or omitted variables in the model. Detailed description of model 1, model 2, VPC and PCV are provided in (Supplementary file).

These models 1 and 2 can even be extended to include more than two health outcomes in a multivariate multilevel model (49, 50). This model explicitly evaluates the covariance (i.e., a measure of joint variability of two random variables) between different social strata and health outcomes which allows us not only to draw conclusions about social group-specific differences but also correlations between health outcomes (49). This is desirable since we will assume that they capture related, though distinct, health constructs.

Limitation of this proposed studies is that datasets which will be used were collected over nine years ago (8, 33). Considering the dynamic nature of slums, a more recent data would have been more informative of the SDoH factors which affect under-five health conditions. Despite this, we expect the findings which will obtained to be of great value since these datasets come from the most recently conducted slum surveys in both Bangladesh and Kenya.

Patient and public involvement
There will be no patient or public involvement in this study, as it is based on secondary data.

State date of the analyses
September 2021

Anticipated end date
March 2022

For peer review only - http://bmjopen.bmj.com/site/about/guidelines.xhtml

Ethics and dissemination

The study will use secondary data from the Nairobi Cross-sectional Survey 2012 (NCSS 2012) and Urban Health Survey (UHS 2013) which excludes any participant identifiers. Ethical approval for the NCSS 2012 study was obtained from the Kenya Medical Research Institute's Ethics Review Committee (8) . For UHS 2013, ethical approval was obtained from the Bangladesh Medical Research Council (BMRC) and the Institutional Review Board (IRB) at the School of Public Health, University of North Carolina at Chapel Hill (33). This work as part of ARISE will be used in shaping actions to improve slum health for under five in Bangladesh and Kenya (54). Finding from these studies will be in published peer reviewed journals and presented in international conferences. Analyses will be presented to policy makers and stakeholders of slum health throughout the course of ARISE project.

Data availability

NCSS 2012 data available from African Population Health Centre (APHRC) microdata portal upon reasonable request https://aphrc.org/microdata-portal/ . The UHS 2013 are publicly available at the website of University of North Carolina dataverse portal upon responsible request https://dataverse.unc.edu

Supplementary materials

Ethics statements

Patient consent for publication

Not required

Twitter: @e_kibuchi

Contribution

EK, LG and AL conceived and designed the study. EK wrote the first draft and revised the protocol. PB, IC, NS, PPH, MIHM, CK, ZQ, LW, LD, RF, TS, BA, VS, SG, SS, IG, JKL, BM, HE, AL, and LG critically revised the manuscript.

Funding

The GCRF Accountability for Informal Urban Equity Hub ('ARISE') is a UKRI Collective Fund award with award reference ES/S00811X/1. The MRC/CSO Social and Public Health Sciences Unit is funded by the Medical Research Council (MC_UU_00022/2) and the Scottish Government Chief Scientist Office (SPHSU17).

For peer review only - http://bmjopen.bmj.com/site/about/guidelines.xhtml

Disclaimer: The funders have no role in study design or writing of the report. The views expressed in this article are those of the authors.

Competing interest: None declared.

Figure 1: A causal diagram showing the direct pathways between children's demographics, child's mother characteristics, head of household demographics, social structure characteristics and under-five health conditions.

For peer review only

For peer review only - http://bmjopen.bmj.com/site/about/guidelines.xhtml

References

1. World Health Organization. A billion voices: listening and responding to the health needs of slum dwellers and informal settlers in new urban settings. World Health Organization; 2005.
2. UN-Habitat. State of the World's Cities 2006/7. New York: United Nations. 2006;292.
3. World Health Organization. A conceptual framework for action on the social determinants of health. 2010.
4. Tarlov AR. Social determinants of health: the sociobiological translation. Health and social organization: Routledge; 2002. p. 87-109.
5. Dovey K, King R. Forms of informality: Morphology and visibility of informal settlements. Built Environment. 2011;37(1):11-29.
6. World Health Organization. Health inequities and their causes: World Health Organisation; 2018 [Available from: https://www.who.int/news-room/facts-in-pictures/detail/health-inequities-and-their-causes.
7. World Health Organization. Our cities, our health, our future: acting on social determinants for health equity in urban settings. Report to the WHO Commission on Social Determinants of Health from the Knowledge Network on Urban Settings. Our cities, our health, our future: acting on social determinants for health equity in urban settings Report to the WHO Commission on Social Determinants of Health from the Knowledge Network on Urban Settings. 2008.
[dataset] 8. APHRC. Population and health dynamics in Nairobi's informal settlements: Report of the Nairobi Cross-sectional Slums Survey (NCSS) 2012. Nairobi: APHRC. 2014(April):1-185. https://aphrc.org/microdata-portal/
9. Satterthwaite D, editor Health in urban slums depends on better local data. International Conference on Urban Health, Manchester, UK; 2014.
10. UNICEF. Children in an Urban World: the State of the World's Children 2012. Retrieved July. 2012;24:2012.
11. United Nations. World urbanization prospects: The 2018 revision: UN: Department of Economic Social Affairs. Population Division; 2019.
12. Rutstein S, Staveteig S, Winter R, Yourkavitch J, editors. Urban child poverty, health, and survival in low-and middle-income countries. 2017 International Population Conference; 2017: IUSSP.
13. UNICEF. Understanding urban inequalities in Bangladesh: a prerequisite for achieving Vision 2021. UNICEF Bangladesh, Dhaka. 2010.
14. APHRC. Population and health dynamics in Nairobi's informal settlements: report of the Nairobi Cross-sectional Slums Survey (NCSS) 2000: African Population and Health Research Center; 2002.
15. Barrett JR. A marked disadvantage: rapid urbanization and mortality of young children in Nigeria. National Institute of Environmental Health Sciences; 2010.
16. Kyobutungi C, Ziraba AK, Ezeh A, Yé Y. The burden of disease profile of residents of Nairobi's slums: Results from a Demographic Surveillance System. Population health metrics. 2008;6(1):1-8.
17. Unger A. Children's health in slum settings. Arch Dis Child. 2013;98(10):799-805.
18. Victora CG, Smith PG, Vaughan JP, Nobre LC, Lombard C, Teixeira AMB, et al. Water supply, sanitation and housing in relation to the risk of infant mortality from diarrhoea. International journal of epidemiology. 1988;17(3):651-4.
19. World Health Organization. Hidden cities: unmasking and overcoming health inequities in urban settings: World Health Organization. Centre for Health Development; 2010.
20. Unger A. Children's health in slum settings. Archives of disease in childhood. 2013;98(10):799-805.
21. Commission on Social Determinants of Health. Closing the gap in a generation: health equity through action on the social determinants of health: final report of the commission on social determinants of health: World Health Organization; 2008.

For peer review only - http://bmjopen.bmj.com/site/about/guidelines.xhtml

22. Green MA, Evans CR, Subramanian SV. Can intersectionality theory enrich population health research? Social Science & Medicine. 2017(178):214-6.
23. López N, Gadsden VL. Health inequities, social determinants, and intersectionality. 2016.
24. Sen G, Iyer A. Who gains, who loses and how: leveraging gender and class intersections to secure health entitlements. Social science & medicine. 2012;74(11):1802-11.
25. Homan P, Brown TH, King B. Structural intersectionality as a new direction for health disparities research. Journal of health and social behavior. 2021;62(3):350-70.
26. Larson E, George A, Morgan R, Poteat T. 10 Best resources on… intersectionality with an emphasis on low-and middle-income countries. Health policy and planning. 2016;31(8):964-9.
27. Evans CR, Leckie G, Merlo J. Multilevel versus single-level regression for the analysis of multilevel information: The case of quantitative intersectional analysis. Soc Sci Med. 2020;245:112499.
28. Merlo J. Multilevel analysis of individual heterogeneity and discriminatory accuracy (MAIHDA) within an intersectional framework. Soc Sci Med. 2018;203:74-80.
29. Fisk SA, Mulinari S, Wemrell M, Leckie G, Vicente RP, Merlo J. Chronic obstructive pulmonary disease in Sweden: an intersectional multilevel analysis of individual heterogeneity and discriminatory accuracy. SSM-population health. 2018;4:334-46.
30. Merlo J, Yang M, Chaix B, Lynch J, Råstam L. A brief conceptual tutorial on multilevel analysis in social epidemiology: investigating contextual phenomena in different groups of people. Journal of Epidemiology & Community Health. 2005;59(9):729-36.
31. Friesen J, Friesen V, Dietrich I, Pelz PF. Slums, space, and state of health—a link between settlement morphology and health data. International journal of environmental research and public health. 2020;17(6):2022.
32. Unger A, Riley LW. Slum health: from understanding to action. PLoS Medicine. 2007;4(10):e295.
[dataset] 33. NIPORT ICfDDR, Bangladesh ,, Evaluation M. Bangladesh urban health survey 2013. Final report. NIPORT, ICDDRB, and MEASURE Evaluation Dhaka, Bangladesh and Chapel Hill …; 2015. https://dataverse.unc.edu
34. Agarwal S, Bhanot A, Goindi G. Understanding and addressing childhood immunization coverage in urban slums. Indian pediatrics. 2005;42(7):653.
35. Ernst KC, Phillips BS. Slums are not places for children to live: vulnerabilities, health outcomes, and possible interventions. Advances in pediatrics. 2013;60(1):53-87.
36. Vaid A, Mammen A, Primrose B, Kang G. Infant mortality in an urban slum. The Indian Journal of Pediatrics. 2007;74(5):449-53.
37. Crocker-Buque T, Mindra G, Duncan R, Mounier-Jack S. Immunization, urbanization and slums–a systematic review of factors and interventions. BMC public health. 2017;17(1):1-16.
38. Hussain A, Ali SK, Kvåle G. Determinants of mortality among children in the urban slums of Dhaka city, Bangladesh. Tropical Medicine & International Health. 1999;4(11):758-64.
39. Egondi T, Oyolola M, Mutua MK, Elung'ata P. Determinants of immunization inequality among urban poor children: evidence from Nairobi's informal settlements. International journal for equity in health. 2015;14(1):1-5.
40. UN-HABITAT. The challenge of slums: global report on human settlements, 2003: UN-HABITAT -United Nations Human Settlements Programme Staff; 2003.
41. Fotso J-C. Child health inequities in developing countries: differences across urban and rural areas. International journal for equity in health. 2006;5(1):1-10.
42. Fry S, Cousins B, Olivola K. Health of children living in urban slums in Asia and the near east: Review of existing literature and data. Washington, DC: Environmental Health Project, US Agency for International Development. 2002.
43. Sverdlik A. Ill-health and poverty: a literature review on health in informal settlements. Environment and Urbanization. 2011;23(1):123-55.

For peer review only - http://bmjopen.bmj.com/site/about/guidelines.xhtml

44. Butala NM, VanRooyen MJ, Patel RB. Improved health outcomes in urban slums through infrastructure upgrading. Social science & medicine. 2010;71(5):935-40.
45. Buigut S, Ettarh R, Amendah DD. Catastrophic health expenditure and its determinants in Kenya slum communities. International journal for equity in health. 2015;14(1):1-12.
46. Xu K, Aguilar A, Carrin G, Evans D, Hanvoravongchai P, Kawabata K, et al. Distribution of health payments and catastrophic expenditures methodology. 2005. World Health Organization, Discussion Paper. 2017(2).
47. Persmark A, Wemrell M, Zettermark S, Leckie G, Subramanian S, Merlo J. Precision public health: mapping socioeconomic disparities in opioid dispensations at Swedish pharmacies by multilevel analysis of individual heterogeneity and discriminatory accuracy (MAIHDA). PloS one. 2019;14(8):e0220322.
48. Evans CR, Williams DR, Onnela JP, Subramanian SV. A multilevel approach to modeling health inequalities at the intersection of multiple social identities. Soc Sci Med. 2018;203:64-73.
49. Baldwin SA, Imel ZE, Braithwaite SR, Atkins DC. Analyzing multiple outcomes in clinical research using multivariate multilevel models. Journal of consulting and clinical psychology. 2014;82(5):920.
50. Snijders TA, Bosker RJ. Multilevel analysis: An introduction to basic and advanced multilevel modeling: sage; 2011.
51. Wagner P, Merlo J, editors. Discriminatory Accuracy of a Random effect in Multilevel Logistic Regression. International Journal of Epidemiology; 2015: Oxford Univ Press Great Clarendon St, Oxford OX2 6DP, England.
52. Wagner P, Merlo J. Measures of discriminatory accuracy in multilevel analysis. Eur J Epidemiol. 2013;28(1):135.
53. Merlo J, Wagner P, Ghith N, Leckie G. An original stepwise multilevel logistic regression analysis of discriminatory accuracy: the case of neighbourhoods and health. PloS one. 2016;11(4):e0153778.
54. ARISE. Accountability and Responsiveness in Informal Settlements for Equity 2021 [Available from: https://www.ariseconsortium.org/arise-a-new-project-to-improve-accountability-for-health-in-urban-informal-settlements/.

For peer review only

For peer review only - http://bmjopen.bmj.com/site/about/guidelines.xhtml

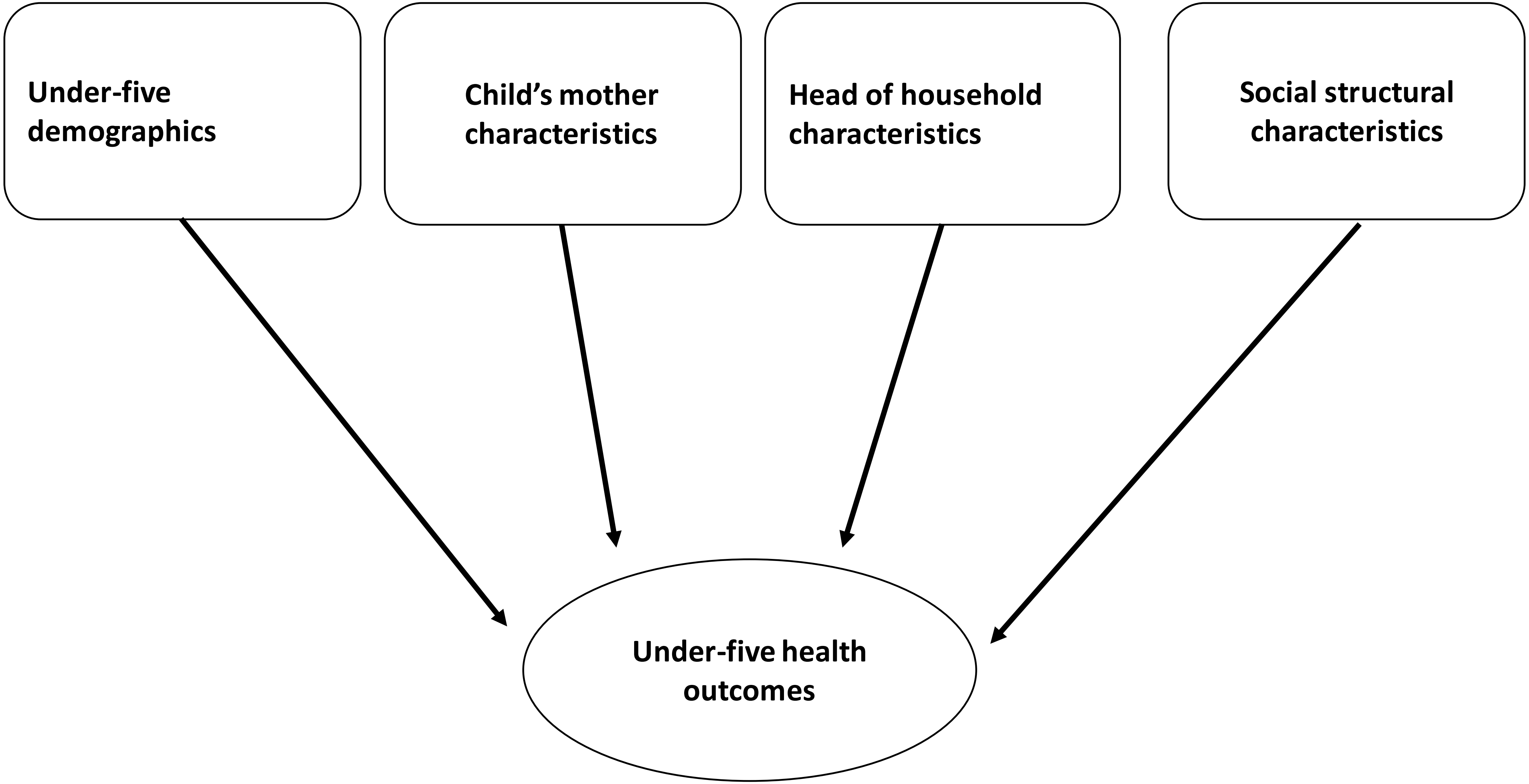
Under-five demographics
Child's mother characteristics
Head of household characteristics
Social structural characteristics
Under-five health outcomes

Supporting information:

Table s1: Description of health outcomes and predictor variables for Nairobi Cross-sectional survey 2012 (NCSS 2012)

Variable name	Description	Categories
**Health outcomes**		
Diarrhea	Whether a child had diarrhea or not in the two weeks preceding the survey	Yes
		No
Fever	Whether a child had fever or not in the two weeks preceding the survey	Yes
		No
Cough	Whether a child had cough or not in the two weeks preceding the survey	Yes
		No
**Predictors**		
**Children demographic characteristics**		
Age	Children up to five were categorised into two groups: “Up to 1 year old (Infants)” and “2-5 years”.	1 year and less (infants)
		2 -5 years
Sex	Sex of a child coded as either male or female	Male
		Female
**Women characteristics**		
Age	Mothers ’age was categorised into two groups: “18 years and less (<18)” and “19 years and above	18 years and under
		19 years and above
**Head of household demographic characteristics**		
Gender	Sex of head of household coded as either male or female	Female
		Male
Ethnicity	Name of ethnic group head of household belongs	Kamba
		Kikuyu
		Luhya
		Luo
		Other
Age	Age of head of household categorised into three categories.	17 – 24years
		25 -34 years
		35 years above
education	Whether head o household has nay education or not	None
		educated
		Don’t know and not applicable

For peer review only - http://bmjopen.bmj.com/site/about/guidelines.xhtml

Social structure		
Wealth index	Wealth Index were regrouped from five (categories) to three by combining poorest and poorer into one group "poor" and richer and richest into one group "rich" and "Middle".	Rich
		Middle
		Poor
Length of stay	The number of years lived in slums by the household grouped into new migrants (i.e.,2 years and less), old migrants (i.e., more than 2 years), and not applicable/missing.	New migrants
		Old migrants
		Not applicable
Household religion	Name of the religion attended by household	Catholic
		Protestant
		Other
Disability in household	Whether a household has any disabled person residing with them.	Yes
		No
		Missing/Not applicable
Tenure	Does the household own or pay rent to the house they live?	No rent paid
		Pays rent
Food availability	Household's availability of any food in the last 12 months	enough
		not enough
Income generating activity	Household's main source of income	Employed
		Own business
		Not applicable
Health Insurance	Whether a household has a health medical insurance (i.e., either public or private or both)	Yes
		No
health catastrophic costs	Did a household face catastrophic health cost at 40% threshold of in the last 30 days	No
		Yes

Table s2: Description of health outcomes and predictor variables for the analysis for Bangladesh Urban Health Survey 2013 (UHS 2013)

Variable name	Description	Categories
**Health outcomes**		
Acute Respiratory Infection (ARI)	Fever is defined as whether a child had fever or not in the two weeks preceding the survey	Yes
		No
Fever	Whether a child had fever or not in the two weeks preceding the survey	Yes
		No
Cough		Yes

For peer review only - http://bmjopen.bmj.com/site/about/guidelines.xhtml

	Whether a child had cough or not in the two weeks preceding the survey	No
**Predictors**		
**Children demographic characteristics**		
Age	Children up to five were categorised into two groups: "Up to 1 year old (Infants)" and "2-5 years".	1 year and less (infants)
		2 -5 years
Sex	Sex of a child coded as either male or female	Male
		Female
**Women characteristics**		
Age	Mothers 'age was categorised into two groups: "18 years and less (<18)" and "19 years and above. Note that the legal age of women at first marriage is 18 years in Bangladesh.	18 years and under
		19 years and above
Ever attended school	Mother ever attended school	Yes
		No
Highest education	Mother's level of education categorised into four groups: "Higher", "Secondary", "Primary", and "No education"	Higher
		Secondary
		Primary
		No education
Marital status	Mother's marital status was categorised into two groups: "Being married", "Not being married"	Being married
		Not being married
Employment	Mothers of respective children was employed last 12 months.	Yes
		No
Religion	Mother's religion was categorized into two groups: "Islam", "Minority religion". Note that Buddhism, Hinduism, Christianism were combined as minority religion in this study.	Islam
		Minority religion
**Head of household demographic characteristics**		
Gender	Sex of head of household was coded as either male or female	Female
		Male
Age	Age of head of household was categorised into three categories.	17 – 24years
		25 -34 years
		35 years above
Marital status	Head of household's marital status was categorised into two groups: "Being married", "Not being married"	None
		educated
		Don't know and not applicable
**Social structure**		
Wealth index	Wealth Index were regrouped from five (categories) to three by combining poorest and poorer into one group	Rich
		Middle

For peer review only - http://bmjopen.bmj.com/site/about/guidelines.xhtml

	"poor" and richer and richest into one group "rich" and "Middle".	Poor
Length of stay	The number of years lived in slums by the household grouped into new migrants (i.e.,2 years and less), old migrants (i.e., more than 2 years), and not applicable/missing.	New migrants
		Old migrants
		Not applicable
Cooking Fuel used in household	Cooking fuels used in the household were categorized into four: "Charcoal, dung cakes etc.", "Kerosene or liquid gas", "Natural gas", and "Wood fuel"	Charcoal, dung cakes etc.
		Kerosene or liquid gas
		Natural gas
		Wood fuel
Garbage disposal method of households	Garbage disposal method of households were categorized into four: "Disposed within premises", "Collected from home", "Disposed in bin outside", and "Disposed in open spaces"	Disposed within premises
		Collected from home
		Disposed in bin outside
		Disposed in open spaces
Migration status of households	Household's availability of any food in the last 12 months	
Housing Type	Housing type was categorized into two groups: "Multiple story" and "Single story". We collapsed *Jhupri, Mess* as single story.	Multiple story
		Single story
Ownership of the dwelling	Two categories: "yes", "no"	No
		Yes
Ownership of the land	Two categories: "yes", "no"	No
		Yes
Having Separate kitchen	Two categories: "yes", "no"	No
		Yes
Division	Administrative divisions were categorized into four groups: "Dhaka", "Khulna", "Rajshahi" and "Others division". Note that "Barisal", "Chittagong", "Rangpur", and "Sylhet" were combined as "Others division" where ARISE Bangladesh Team conducts research.	Others division
		Dhaka
		Khulna
		Rajshahi

Statistical methodology details

As an illustrative example, consider investigating the effects of child's age (i.e., up to 1 year, 2 to 3 years., 4 to 5 years), child's sex (i.e., female or male), head of household education (none, primary, secondary and higher), sex of the head of household (i.e., male or female), age of the head of household (i.e., 18-25 years, 26-32 years, 33-40 years, >40 years) and household's health insurance status (yes or no). Using the six variables and their corresponding categories: child's age (3), child's sex (2), head of household education (3), head of household sex (2), head of household age (4) and

For peer review only - http://bmjopen.bmj.com/site/about/guidelines.xhtml

household's health insurance status (2) we can create 288 intersectional groups/strata. The first stratum can consist of a child who is a female, aged up to 1 year and less, coming from a household whose head is a female, aged 18-25 years and with no education and the household is not covered by health insurance, and the process continues until all the children at level 1 are nested within the groups/strata $(N = 288)$ at level 2. This implies that children at level 1 who share similar SDoH factors end up being in the same intersectional group/stratum at level 2.

To capture differences in health disparities in children health outcomes between different groups, for example - males and females, we can assess child's sex-specific effects through their interactions with child 's age, head of household's sex, age and education and household's health insurance using interaction-based fixed model. However, interaction -based fixed effects model formulation only addresses interactions between child's sex and each of the other five variables and not all interactions Eq. 3.

If we were to consider all possible interactions among the six variables, we will have 288 interaction terms in the regression model which may result in the model having parsimony and scalability issues due to geometrical growth of coefficients as more variables are included in the model. In addition, it would be difficult to interpret the results and reduced sample size in some interaction groups may influence whether an association is determined or not (Goldstein, 2011; J. Merlo, 2018; *Supplemental file*). We will overcome this limitation of interaction-based fixed effects models by applying MAIHDA approach (Snijders & Bosker, 2011). This involves treating social strata/groups defined by child's age and sex, head of household sex, age and education and household's health insurance status as strata which will used to explain whether health inequalities are shaped by different characteristics in each stratum.

Let us consider the case where we are interested in investigating effects of child's age (i.e., up to 1 year and less, 2 to 3 years., 4 to5 years), child's sex (i.e., female or male), head of household sex (i.e., male or female), head of household education (none, primary, secondary and higher), head of household age (i.e.,18-25 years, 26-32 years, 33-40 years., >40 years) and household health insurance (yes or no) on children's diarrhea in slums.

Let y_i denote a health outcome of interest (i.e., diarrhea) for child $i(i = 1, \dots, i)$ where,

$$y_i = \begin{cases} 0 \ \ absence\ of\ diarhea \\ 1 \ \ Presence\ of\ diarrhea \end{cases} \quad \text{Eq.(1)}$$

y_i is assumed to follow a Bernoulli distribution, with probabilities $\pi_i = Pr(y_i = 0)$ the probability of child i having no diarrhea and $1 - \pi_i = Pr(y_i = 1)$ the probability of child i having diarrhea. Let X_{1i}represent child sex, X_{2i} represent child's age, X_{3i}represent head of household sex, X_{4i} represent

For peer review only - http://bmjopen.bmj.com/site/about/guidelines.xhtml

head of household education, X_{5i} represent head of household age and X_{6i} represent household health insurance. These six variables represent predictor variables. Logistic regression is appropriate for modelling binary (two category) outcomes such as whether a child has diarrhea or not.

The fixed effects logistic regression model for investigating how child sex, child age, head of household sex, head of household education, head of household age and household health insurance are additively associated with child's diarrhea is represented in equation 1 "Eq. (2)".

$$log\left(\frac{\pi_i}{1-\pi_i}\right) = \beta_0 + \beta_1 X_{1i} + \beta_2 X_{21i} + \beta_3 X_{22i} + \beta_4 X_{3i} + \beta_5 X_{41i} + \beta_6 X_{42i} + \beta_7 X_{51i} + \beta_8 X_{52i} + \beta_9 X_{53i} + \beta_{10} X_{6i} \quad \text{Eq. (2)}$$

Eq. (2) estimates the associations of child sex, child age, head of household sex, head of household education, head of household age and household health insurance with child's diarrhea additively (i.e., explanatory effects) and does not accommodate for interactions with each other. In order, to capture specific effects between different groups, for example, child's sex (i.e., males or females), we can assess sex-specific disparities in diarrhea through their interactions with child age, head of household sex, head of household education, head of household age and household health insurance.

Eq. (2) can thus be expanded to include interaction terms, as presented as follows in Eq. (3):

$$log\left(\frac{\pi_i}{1-\pi_i}\right) = \beta_0 + \beta_1 X_{1i} + \beta_2 X_{21i} + \beta_3 X_{22i} + \beta_4 X_{3i} + \beta_5 X_{41i} + \beta_6 X_{42i} + \beta_7 X_{51i} + \beta_8 X_{52i} + \beta_9 X_{53i} + \beta_{10} X_{6i} + \beta_{11} X_{21i} X_{1i} + \beta_{12} X_{22i} X_{1i} + \beta_{13} X_{3i} X_{1i} + \beta_{14} X_{41i} X_{1i} + \beta_{15} X_{42i} X_{1i} + \beta_{16} X_{51i} X_{1i} + \beta_{17} X_{52i} X_{1i} + \beta_{18} X_{53i} X_{1i} + \beta_{19} X_{6i} X_{1i} \quad \text{Eq. (3)}$$

whereβ_{11} to β_{19} are interaction coefficients between child's sex and other explanatory variables in Eq. (2). Not only does Eq. (3) allow for an analysis that considers the association of child's sex in getting diarrhea but also uncovers how other factors that create and sustain diarrhea may differ based on the sex of child. However, Eq. (3) only addresses interactions between child's sex and the other five variables and if we were to consider all possible interactions among the six variables, we will have a total of 288 fixed effects in the logistic model. The higher number of fixed effects may lead to issues with scalability (i.e., a model's inability to accommodate an increase in the number of variables included), model parsimony (i.e., a simple model not having great explanatory predictive power) and reduced sample size in some intersectional groups which may influence whether an effect is determined is determined or not. In addition, it would be difficult to interpret 288 fixed effects. We can overcome these issues by using multilevel analysis of individual heterogeneity and discriminatory accuracy (MAIHDA) approach.

For peer review only - http://bmjopen.bmj.com/site/about/guidelines.xhtml

Multilevel analysis of individual heterogeneity and discriminatory accuracy (MAIHDA)

Now, let consider the case where we are interested in investigating effects child's age (i.e., up to 1 year and less, 2 to 3 years., 4 to5 years), child's sex (i.e., female or male), head of household sex (i.e., male or female), head of household education (none, primary, secondary and higher), head of household age (i.e.,18-25years, 26-32years, 33-40 years, >40years) and household health insurance (yes or no) on the outcome diarrhea via intersectionality lens using MAIHDA (Evans, Leckie, & Merlo, 2020; J. Merlo, 2018). This can procced in 3 steps:

The first step involves creating groups/strata based on the categories of the social determinants of health factors (SDoH) we are interested in. This means that children at level 1 who share similar categories SDoH will end up being in the same group/strata at level 2. Therefore, in this example we will have individuals at level 1 nested within 288 groups at level 2.

Therefore, let y_{ij} denote a binary health outcome (i.e., diarrhea) for child $i(i = 1, \dots, n)$ in groups $j\ (j = 1, \dots, N)$ where,

$$y_{ij} = \begin{cases} 0 & absence\ of\ diarrhea \\ 1 & Presence\ of\ diarrhea \end{cases} \quad \text{Eq.(4)}$$

y_{ij} is assumed to follow a Bernoulli distribution, with probabilities $\pi_{ij} = Pr(y_{ij} = 0)$ the probability of child i from stratum/group j having no diarrhea and $1 - \pi_{ij} = Pr(y_i = 1)$ the probability of child i from stratum/group j having diarrhea. Let X_{1ij} represent child sex, X_{2ij} represent child's age, $X_{3ij}j$represent head of household sex, X_{4ij} represent head of household education, X_{5ij} represent head of household age and X_{6ij} represent household health insurance.

The next step involves fitting a null model with children at level one nested within social groups at level two to assess whether there is significant clustering within intersectional strata/groups constructed in step 1. The null model will not include any predictor variables and will only have an intercept to estimate the mean health condition and a random effect to model intersectional strata differences (i.e., variance) and is presented in Eq. 5

$$log\left(\frac{\pi_{ij}}{1-\pi_{ij}}\right) = \beta_0 + \mu_{0j} \quad \text{Eq. (5)}$$

where β_0 is the intercept and $\mu_{0j} \sim N(0, \sigma^2_{group})$ represents the random intercept for the intersectional stratum level residual which is normally distributed with mean 0 and variance σ^2_{μ}. Eq. (5) includes no predictor variables, so the intersectional stratum random effect captures both the main effects of SDoH used to define intersectional strata and their interactions. Assuming no omitted variable bias, the intersectional strata level residual μ_{0j} captures the unique interaction effect for

For peer review only - http://bmjopen.bmj.com/site/about/guidelines.xhtml

each intersectional strata (i.e., intersectional -specific differences in health condition) while accounting for sample size differences for each social group. The relevance of the intersectional strata for understanding individual heterogeneity will be evaluated using Variance partitioning Coefficient (VPC) also known as intraclass coefficient (which also informs on the discriminatory accuracy of the intersectional categorisation for distinguishing children with diarrhea from those without (Juan Merlo, Yang, Chaix, Lynch, & Råstam, 2005; Wagner & Merlo, 2013). VPC will be used to quantify the share of the total individual variance in having a health condition that is accounted for at the intersectional strata level with values higher than 5% indicating an acceptable DA (Fisk et al., 2018; Wagner & Merlo, 2013). That is, a high VPC indicates that intersections have a substantially different mean levels of an outcome and that individuals within these group are similar, while a low VPC indicates that individuals within an intersectional group differ substantially. On the other hand, AUC-ROC measures the ability of the model to classify individuals with or without health outcome as a function of individual's predicted probabilities and is and is bounded between 0.5 and 1 (Fisk et al., 2018; Wagner & Merlo, 2013). A value of 0.5 indicates that model predictions are no better than random guessing meaning that predictor variables used in the model have no predictive power, while a value of 1 represents perfect discrimination between under-five with or without health condition (Fisk et al., 2018; Wagner & Merlo, 2013). In our proposed analyses, AUC-ROC values greater than 0.7 and VPC greater than 5% will indicate an acceptable DA and existence of intersectional effects.

The next step involves extending Eq. (5) by can be extended into Eq. (6) by by adjusting for variables used in constructing intersectional strata as fixed effects. Therefore Eq. (6) takes the form:

$$log\left(\frac{\pi_{ij}}{1-\pi_{ij}}\right) = \beta_0 + \beta_1 X_{1ij} + \beta_2 X_{21ij} + \beta_3 X_{22ij} + \beta_4 X_{3ij} + \beta_5 X_{41ij} + \beta_6 X_{42ij} + \beta_7 X_{51ij} + \beta_8 X_{52ij} + \beta_9 X_{53ij} + \beta_{10} X_{6ij} + \mu_{0j} \quad \text{Eq. (6)}$$

where β_0 is the intercept and $\mu_{0j} \sim N\left(0, \sigma^2_{group}\right)$ represents the group level residual which is normally distributed with mean 0 and variance σ^2_{group}. Assuming no omitted variable bias, the group level residual μ_{0j} captures the unique interaction effect for each social group/strata (i.e. social groups -specific differences in diarrhea) while accounting for sample size differences for each social group. Eq (6) will be used to explore to which extent intersectional strata differences will be explained by SDoH used in constructing intersectional groups. The proportion of variance explained by the adding fixed effects is estimated by calculating the proportional change in variance (PCV) of intersectional strata between a null model defined by Eq. (5) and model with fixed effects represented by Eq. (6) (Wagner & Merlo, 2013, 2015). The lower the PCV, the higher the amount of unexplained variance which can be due to either interaction effects or omitted variables in the model.

For peer review only - http://bmjopen.bmj.com/site/about/guidelines.xhtml

References

Evans, C. R., Leckie, G., & Merlo, J. (2020). Multilevel versus single-level regression for the analysis of multilevel information: The case of quantitative intersectional analysis. *Soc Sci Med, 245*, 112499. doi:10.1016/j.socscimed.2019.112499

Fisk, S. A., Mulinari, S., Wemrell, M., Leckie, G., Vicente, R. P., & Merlo, J. (2018). Chronic obstructive pulmonary disease in Sweden: an intersectional multilevel analysis of individual heterogeneity and discriminatory accuracy. *SSM-population health, 4*, 334-346.

Goldstein, H. (2011). *Multilevel statistical models* (Vol. 922): John Wiley & Sons.

Merlo, J. (2018). Multilevel analysis of individual heterogeneity and discriminatory accuracy (MAIHDA) within an intersectional framework. *Soc Sci Med, 203*, 74-80. doi:10.1016/j.socscimed.2017.12.026

Merlo, J., Yang, M., Chaix, B., Lynch, J., & Råstam, L. (2005). A brief conceptual tutorial on multilevel analysis in social epidemiology: investigating contextual phenomena in different groups of people. *Journal of Epidemiology & Community Health, 59*(9), 729-736.

Snijders, T. A., & Bosker, R. J. (2011). *Multilevel analysis: An introduction to basic and advanced multilevel modeling*: sage.

Supplemental file. Retrieved from

Wagner, P., & Merlo, J. (2013). Measures of discriminatory accuracy in multilevel analysis. *Eur J Epidemiol, 28*(1), 135.

Wagner, P., & Merlo, J. (2015). *Discriminatory Accuracy of a Random effect in Multilevel Logistic Regression.* Paper presented at the International Journal of Epidemiology.

For peer review only
